# Building blocks of microphysiological system to model physiology and pathophysiology of human heart

**DOI:** 10.3389/fphys.2023.1213959

**Published:** 2023-07-06

**Authors:** Hanna Vuorenpää, Miina Björninen, Hannu Välimäki, Antti Ahola, Mart Kroon, Laura Honkamäki, Jussi T. Koivumäki, Mari Pekkanen-Mattila

**Affiliations:** ^1^ Centre of Excellence in Body-on-Chip Research (CoEBoC), BioMediTech, Faculty of Medicine and Health Technology, Tampere University, Tampere, Finland; ^2^ Adult Stem Cell Group, Faculty of Medicine and Health Technology, Tampere University, Tampere, Finland; ^3^ Research, Development and Innovation Centre, Tampere University Hospital, Tampere, Finland; ^4^ Micro- and Nanosystems Research Group, Faculty of Medicine and Health Technology, Tampere University, Tampere, Finland; ^5^ Computational Biophysics and Imaging Group, Faculty of Medicine and Health Technology, Tampere University, Tampere, Finland; ^6^ Biomaterials and Tissue Engineering Group, Faculty of Medicine and Health Technology, Tampere University, Tampere, Finland; ^7^ Neuro Group, Faculty of Medicine and Health Technology, Tampere University, Tampere, Finland; ^8^ Heart Group, Faculty of Medicine and Health Technology, Tampere University, Tampere, Finland

**Keywords:** cardiac modeling, microphysiological systems, *in vitro*, *in silico*, co-cultures, biomaterials, imaging, environmental control

## Abstract

Microphysiological systems (MPS) are drawing increasing interest from academia and from biomedical industry due to their improved capability to capture human physiology. MPS offer an advanced *in vitro* platform that can be used to study human organ and tissue level functions in health and in diseased states more accurately than traditional single cell cultures or even animal models. Key features in MPS include microenvironmental control and monitoring as well as high biological complexity of the target tissue. To reach these qualities, cross-disciplinary collaboration from multiple fields of science is required to build MPS. Here, we review different areas of expertise and describe essential building blocks of heart MPS including relevant cardiac cell types, supporting matrix, mechanical stimulation, functional measurements, and computational modelling. The review presents current methods in cardiac MPS and provides insights for future MPS development with improved recapitulation of human physiology.

## 1 Introduction

Microphysiological systems (MPS) are advanced *in vitro* platforms for modeling human or animal organ and tissue functions in health and in disease. MPS possess great potential as alternatives to animal models in basic research, drug development and toxicology. The United States Food and Drug Administration (FDA) defines MPS as “a microphysiological system which uses microscale cell culture platform for *in vitro* modeling of functional features of a specific tissue or organ of human or animal origin by exposing cells to a microenvironment that mimics the physiological aspects important for their function or pathophysiological condition”.^1^ This also requires controlling and monitoring the cellular microenvironment and cellular heterogeneity of the tissues. MPS development, production, and utilization require collaboration across various scientific fields, including cell biology, biomaterials science, microfabrication, sensor technology, signal processing and computer modeling.

Probably due to the rapid development^1^ of the field, MPS is an inclusive term encompassing also body-on-chips and organ-on-chips. While these devices share similarities, organ-on-chips are a subset of MPS with microfluidic cell cultures especially suitable to recapitulate tissue–tissue interfaces (Abulaiti et al., 2020; Kavand et al., 2022).

**FIGURE 1:**
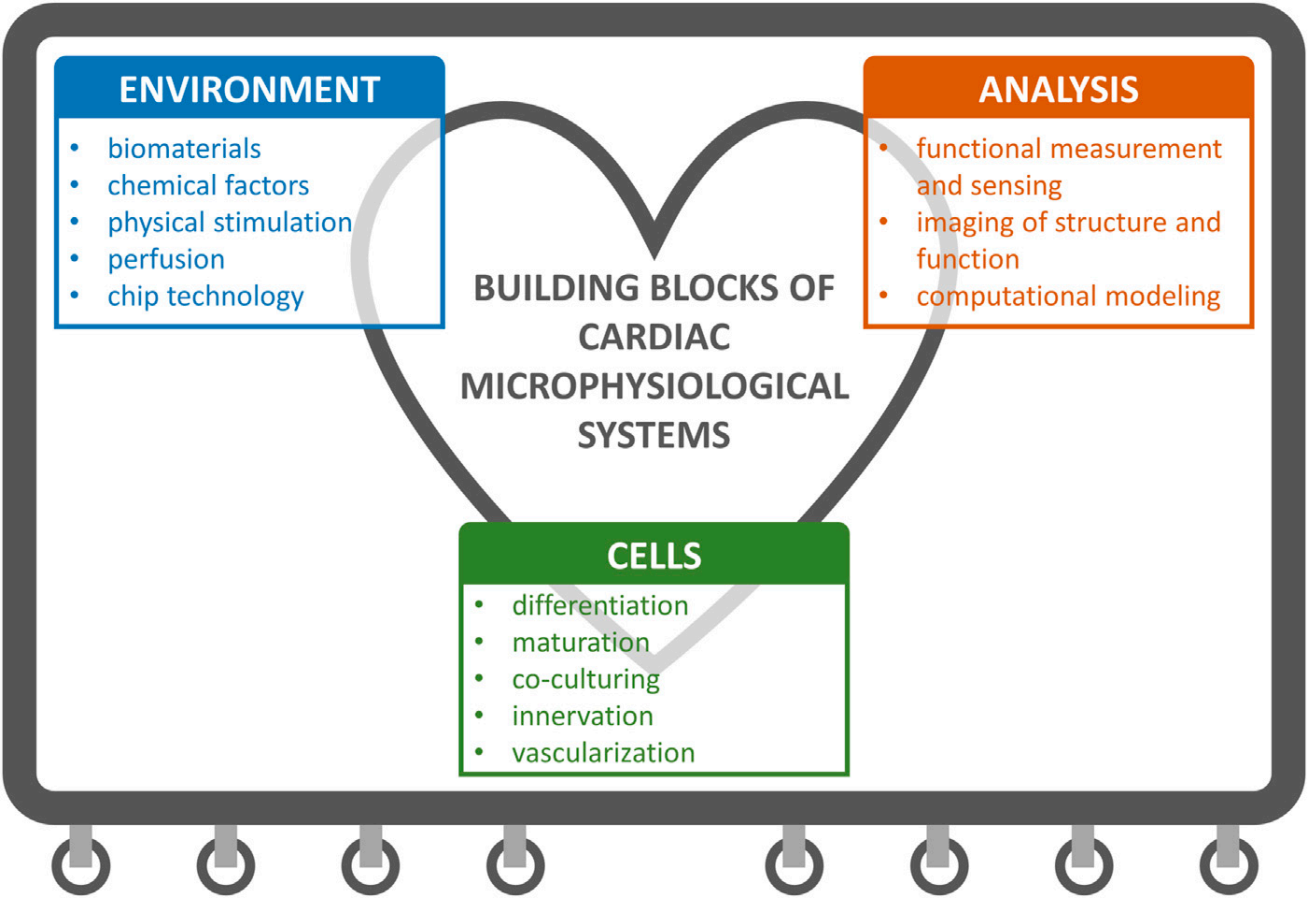
Building blocks of cardiac microphysiological systems (MPS). We cover the topics that fall under these three main categories in the review.

Important aspects when developing MPS encompass three main areas illustrated in Figure 1: 1) cells that enable clinically and physiologically relevant model), 2) environment for supporting and controlling the cell behavior, and 3) means to analyze, monitor and predict the physiological model. In this review, we propose a block-wise approach is for MPS development, where the building blocks are selected according to the specific research questions and applications.

Although, human induced pluripotent stem cell (hiPSC)-based cells are commonly used in MPS, animal-based, primary cells or cancer cell lines, cell explants or organoids can also be utilized depending on the research questions. Cell environment plays a crucial role in cell behavior including maturation. Traditional 2D cell culture platforms have a limited capacity to be used as a model for cardiac diseases and therefore more advanced tissue models have been developed.

MPS rely heavily on material engineering. Hydrogels are commonly used as ECM mimicking supporting matrices for 3D cell cultures. As part of MPS, the hydrogels not only need to provide physical and chemical cues for the cells but they need to have suitable rheological and *in situ* gelation properties for being injected into the systems. During culture, controlling or preventing shrinkage and degradation, and withstanding shear stress becomes important along with other aspects discussed in the coming chapters.

At the core of MPS lies a microfluidic device composed of channels and chambers. In addition, MPS may comprise also micropumps or valves, sensors, and electrodes (Kavand et al., 2022). Polydimethylsiloxane (PDMS) is a commonly used elastomer in MPS fabrication due to its biocompatibility, optical transparency, low autofluorescence, high elasticity, excellent oxygen permeability, and deformability (Radisic and Loskill, 2021; Cao et al., 2023). However, PDMS has the drawback of absorbing molecules, potentially leading to misinterpretation of drug efficacy and toxicity studies. Other elastomers, as well as, glass, thermoplastics and epoxy resins have been employed in MPS fabrication, using methods like 3D printing, lithography, molding, injection molding, casting, and laser cutting as reviewed in (Campbell et al., 2021; Tajeddin and Mustafaoglu, 2021).

This review describes different building blocks of MPS to model physiology and pathophysiology of the human heart. Focus areas include current methods to improve cardiac maturation, state-of-the-art cellular co-cultures, *in vitro* vascularization, *in vitro* innervation, biomaterials, *in silico* approaches for cardiac maturation, and environmental control. The review presents current methods for cardiac modeling in controlled environment and provides insights for development of more accurate models with improved recapitulation of the human native cardiac tissue.

The review is organized as follows:

• Chapter 2 introduces the unmet need for human-based cardiac models.
• Chapter 3 provides a brief overview of the phenotypic peculiarities of hiPSC-CMs and engineered heart tissue (EHT), highlighting the role of MPS in promotion and assessment of their maturation.
• Chapter 4 summarizes the cellular composition and cell-cell interactions in the cardiac MPS with vascularization and innervation.
• Chapters 5 reviews advancements in physical stimulation and biomaterial approaches.
• Chapters 6 and 7 outline the recent development in control and measurement techniques, as well as in computational modeling.
• Chapter 8 delineates possible directions for future work to empower MPS.

The authors of this review include researchers working in the *Centre of Excellence in Body-on-Chip Research* (CoEBoC) in Tampere University, Tampere, Finland. Each author provides insights into their respective areas of expertise, discussing the current state and prospects of different MPS building blocks.

## 2 Unmet needs for human cardiac models and role of MPS

One major application for cardiac *in vitro* models is cardiotoxicity assessment as a part of safety and efficacy studies in drug development. In EU, 312,000 animals, mainly mice, rats and pigs, were used for basic research on cardiovascular, blood and lymphatic system studies at 2019 (Animals in science - European Commission, n.d.). Furthermore, 65,000 animals were used for translational research on human cardiovascular diseases (Animals in science - European Commission, n.d.). However, there are critical differences in physiology of humans and other animals. For instance, mice have a resting heart rate of ∼600 beats per minute (BMP) while in humans it is approximately 60 BMP. At the cell level, mouse hearts have different ion channels that define the basic unit of electrical activity, i.e., the action potentials (APs), different machinery for handling calcium, and different structural protein isoforms, e.g., in myosin (Greenberg et al., 2018). The differences in AP shape are mainly due to species-specific expression levels of repolarizing potassium ion (K^+^) channels (Bartos et al., 2015). Importantly, mouse cardiomyocytes lack the hERG channel (Greenberg et al., 2018). hERG-mediated current plays a major role in human cardiac repolarization and its inhibition, e.g., by certain drugs, is a common cause of severe ventricular arrhythmias such as Torsades-des-Pointes. Thus, evaluation of hERG toxicity has been strongly recommended by the regulatory agencies such as FDA and European Medicines Agency.

For cardiotoxicity assessment but also for other applications predicting human responses, it is essential to use test systems based on human physiology. The FDA workshop “Improving Cardiotoxicity Assessment with Human-Relevant Platforms” concluded that human cell-based models can assess both structural and functional abnormalities of cardiomyocytes and may avoid false positives due to animal-specific findings. A cross-company study by Ewart et al. (2014) on a concordance between conscious dog telemetry data and phase I human cardiovascular assessment showed that although detection of test compounds affecting to corrected QT interval was good within a certain exposure range, the predictive value for heart rate and diastolic blood pressure was poor. More precisely, the unadjusted positive predictive value ranges from dogs to humans was only 10%–13% for heart rate and 6%–8% for diastolic blood pressure with several false positive findings. Human induced pluripotent stem cell derived cardiomyocytes (hiPSC-CM) have shown potential to identify delayed responses, reveal patient-specific susceptibilities, and detect some off-target effects. The workshop, however, emphasized that adult human primary CMs could be considered as the gold standard with full complement of ion channels, receptors, and contractile machinery for cell-based cardiac toxicity studies (Pang et al., 2019). More recent announcement from FDA stated that, according to “Modernization Act 2.0”, mandatory animal experiments are no longer required in drug development before entering clinical trials and the non-clinical test systems can include human relevant cell-based assays, MPS or bioprinted models, or computer models. Beside the on-going paradigm shift in regulatory testing requirements, predictive MPS are needed in revealing (patho-)physiological mechanisms of human tissues with the possibility to study, for the first time, systemic effects.

Since their invention, the hiPSC-CMs have been utilized extensively in genetic cardiac disease modeling, such as modeling long QT-syndrome type 2 and Brugada syndrome (Lahti et al., 2012; Malan et al., 2016). Most of the disease modeling is performed in 2D environment that restricts cellular interaction to a few planar cell–cell crosstalk on a stiff surface. More advanced platforms allowing 3D structure have the potential to overcome the shortcomings of 2D cell culture and provide physiologically more relevant tissue models. One example of such are engineered heart tissues (EHTs). They are 3D-constructs composed of cardiomyocytes with or without the use of scaffold materials, providing mechanical load and support to the tissue construct (Li and Zhang, 2017). EHTs recapitulate normal tissue organization and enable also the assessment of heart muscle function and force generation (Weinberger et al., 2017; Ronaldson-Bouchard et al., 2018; Goldfracht et al., 2019). Another emerging technique possessing potential to be used in cardiac disease modeling are organoids. These multicellular self-assembled structures provide 3D model with morphological complexity including chamber formation, atrioventricular specification, electrophysiological activity as well as vascular structures (Lewis-Israeli et al., 2021).

hiPSC-CMs provide outstanding opportunities for production of cardiac disease models. However, when increasing the complexity level of hiPSC-based cardiac tissue cultures (e.g., 3D-organoids or EHTs) also the functional assessment of the cells becomes more challenging. Therefore, it is suggested that the MPS can be accessorized according to the disease modeling needs; some applications can utilize 2D single cells whereas others require more tissue-like structures and more advanced functional assessment methods.

## 3 Characteristics of hiPSC derived cardiomyocytes

Human pluripotent stem cells have been successfully differentiated into cardiomyocytes (CMs) for over 20 years. First differentiation protocols utilized spontaneous differentiation in embryoid bodies (EBs) (Kehat et al., 2001). Since then, more defined methods have been developed which are based on growth factors and small molecules as recently reviewed (Lyra-Leite et al., 2022). In addition to the traditional differentiation methods from hiPSCs, direct differentiation provides intriguing possibilities especially for regenerative medicine applications, where cardiac fibroblasts in the failing heart can be directly differentiated into functional CMs. Currently, direct reprogramming using transcription factors and microRNAs remains uncontrolled and inefficient (Andrianto et al., 2023).

Despite advancements in hiPSC-CM differentiation, the obtained CMs do not resemble native adult human CMs but exhibit a fetal phenotype which limits their utilization. Native human ventricular CMs are large, rod-like cells with high length-to-width ratio: 100–150 μm to 20–30 µm. In addition, sarcomere length for human CMs is reported to be 1.68–2.0 µm (Severs, 2000; Tracy and Sander, 2011). The hiPSC-CMs do not resemble this highly organized structure, they are much smaller in size, and the sarcomere structure is unorganized (Ahmed et al., 2020). Another notable difference is the absence of T-tubulus structures in hiPSC-CMs, which are invaginations of the plasma membrane. They enable the AP to travel to the interior of CMs, which then triggers the synchronized release calcium ions (Ca^2+^) in close proximity to the sarcomeres and initiates contraction (Guo and Pu, 2020). The cardiac AP is composed of complex interplay of multiple cellular ion channels and there are also several receptors that control the cytoplasmic Ca^2+^ concentration. This functionality is not similar in hiPSC-CMs and the adult counterparts, resulting in different electrophysiological biomarkers. In contrast to their native counterparts and, the hiPSC-CMs also exhibit automaticity. The presence of pacemaker channels and spontaneous intracellular Ca2+ release lead to spontaneous depolarization of the membrane potential and triggering of an AP (Kim et al., 2015).

The sarcomere contraction-relaxation and Ca^2+^ oscillations have been estimated to consume approximately 6 kg of ATP daily in adult human heart (Neubauer, 2007). Due to this high energy consumption rate, the mitochondria content of mature CMs is high and they are estimated to occupy approximately 25% of the cell volume (Barth et al., 1992). Furthermore, the mitochondria in adult CMs are well organized and aligned between myofibrils and under the sarcolemma, whereas mitochondria in hiPSC-CMs are small and distributed throughout the cytoplasm including the perinuclear space (Saks et al., 2012; Karbassi et al., 2020). The hiPSC-CMs use glycolysis as their main energy source whereas native human CMs generate most of their energy from mitochondrial oxidative phosphorylation (Ulmer and Eschenhagen, 2020). To increase the maturation status of hiPSC-CMs, multiple protocols have been published to shift the anaerobic glycolysis-dependent metabolism into aerobic β-oxidation by modifying the culture medium composition (Hu et al., 2018; Horikoshi et al., 2019). Besides maturation, the unique energy metabolism of CMs has been utilized also in the enrichment of the differentiated hiPSC-CMs (Tohyama et al., 2013). Interestingly, non-cardiomyocytes (non-CMs) produced in the hiPSC-CM differentiation process can enhance hiPSC-CM maturation and functionality although the underlying mechanisms behind this cellular communication are not yet fully understood (Kim et al., 2010; Biendarra-Tiegs et al., 2020).

**FIGURE 2:**
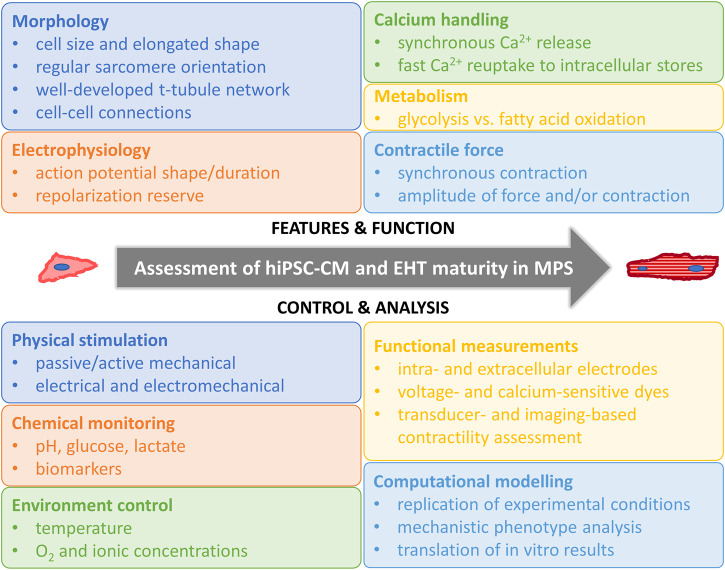
Assessment of hiPSC-CM and EHT maturity in MPS. Human induced pluripotent stem cell derived cardiomyocyte (hiPSC-CM), engineered heart tissue (EHT), microphysiological systems (MPS).

Regardless of the afore-mentioned limitations, hiPSC-CMs hold potential in modeling human cardiomyocytes in cardiac tissue engineering. At the early stages of hiPSC-CMs, they were used in disease modeling as single cells in 2D cultures (Lahti et al., 2012; Pölönen et al., 2018). However, after years of intensive research it is clear that to reliably mimic the human heart and CMs, more advanced culture conditions are required to increase the maturation level of hiPSC-CMs. As reported by Ronaldson-Bouchard, matured iPS-derived cardiac tissue models recapitulated the clinical outcomes of several drugs, such as the bradycardic effects of calcium channel blockers seen in patients (Ronaldson-Bouchard et al., 2022). On the contrary, screening of these drugs by using hiPSC-CM 2D cultures revealed tachycardic responses (Zeng et al., 2019). This finding further supports the need for the proper maturation status of cardiac tissue models and development of cardiac MPS as recently reviewed (Ottaviani et al., 2023). More advanced cardiac models will also enable more complex disease phenotype manifestation and assessment, as, for example, in cardiac hypertrophy (Prondzynski et al., 2019). Computer modeling is one emerging technique in cardiac disease modeling that can be utilized in translation of the results gained from hiPSC-CMs to humans (Paci et al., 2015; Koivumäki et al., 2018). Some key features of hiPSC-CM maturity and ways to measure them are summarized in Figure 2.

## 4 Cellular building blocks and cell-cell interactions in heart MPS

Currently, most of the studies in cardiac MPS are performed using CM single cell cultures from varying CM sources and often without specifying the cardiac cell type in question. Successful cardiac *in vitro* tissue engineering requires cell-cell interactions and paracrine communication between CMs and other cell types naturally present in human heart. In this chapter, we will summarize some key cell types in human heart, their interaction and essential functional characteristics with CMs. Additionally, we survey recently reported methods to build more complex tissue structures leading to vascularized and innervated cardiac *in vitro* models.

**FIGURE 3:**
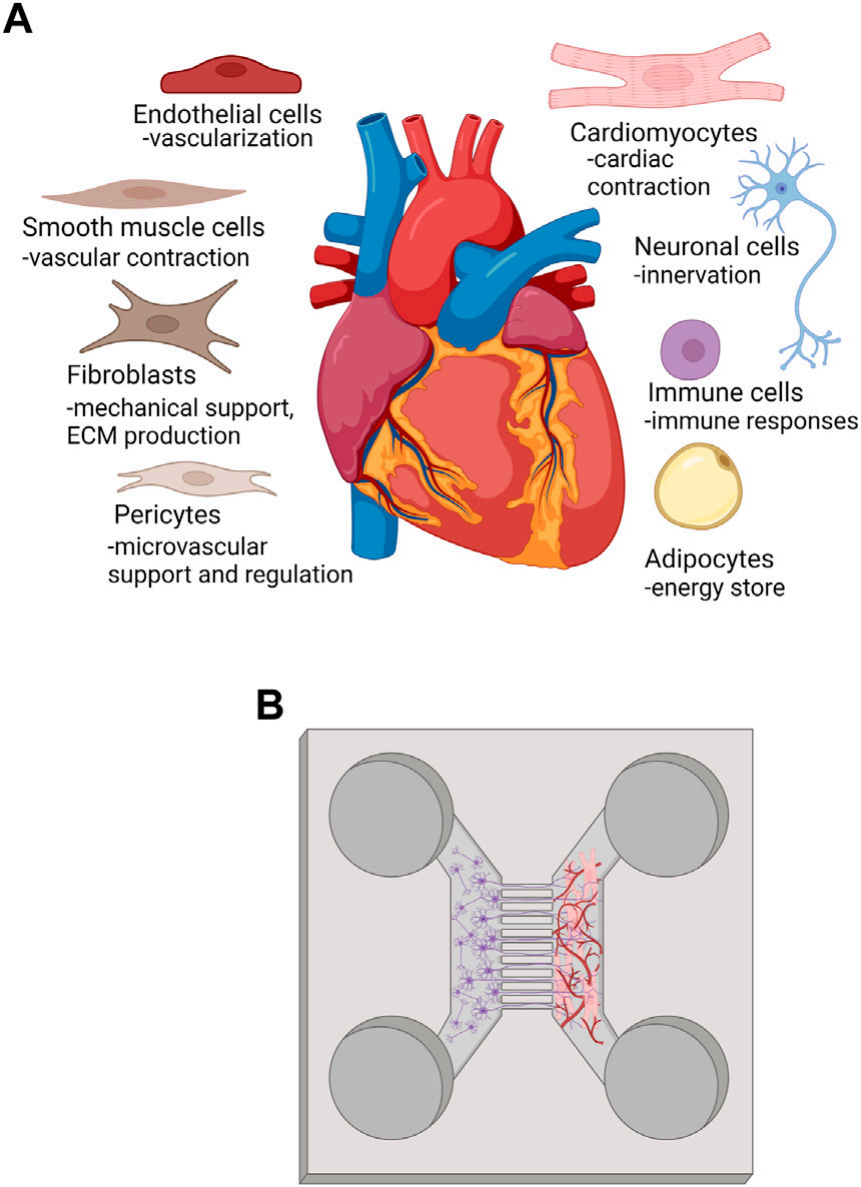
**(A)** main cell types and their functions in human heart, **(B)** representation of a method to innervate and vascularize heart MPS. Created with BioRender.com.

During *in vivo* development, mechanical stimuli, electrical stimuli, extracellular matrix interactions, and non-CM interactions synergistically, and in spatiotemporal manner, coordinate CM maturation process from immature fetal CM stage to functionally mature adult CM phenotype (Scuderi and Butcher, 2017). The heart has considerable regional heterogeneity in the cellular composition regarding resident main cell types. Transcriptomic study of adult human heart cellular composition revealed eleven main cell types including atrial and ventricular cardiomyocytes, fibroblasts, endothelial cells (ECs), pericytes, smooth muscle cells, myeloid and lymphoid immune cells, adipocytes, mesothelial cells and neuronal cells (Figure 3A). More precisely, ventricular tissue consists of approximately 49% CMs, 21% mural cells (pericytes and smooth muscle cells), 16% fibroblasts, 8% ECs and 5% immune cells. CMs are the major cardiac cell type and reside more abundantly in ventricles than in atria, and, interestingly, more in female compared to male ventricular tissue (Litviňuková et al., 2020). Additionally, a small population of resident multipotential cardiac stem/progenitor cells exist in adult human myocardium (Beltrami et al., 2003; Messina et al., 2004). CMs, stromal cells, ECs and other cell types communicate with each other via paracrine signaling, electrical and mechanical coupling, and via interactions with extracellular matrix (ECM). Well-known mechanism in direct cell-cell interaction in the myocardium is connexin (Cx) mediated gap junctional communication that connects the cytoplasm of the interacting cells, enables intercellular exchange of small regulatory molecules and metabolites, and is essential for electrical impulse propagation (Zhang P. et al., 2012). Paracrine intercellular communication is mediated mainly via basic fibroblast growth factors (bFGF), and vascular endothelial growth factor (VEGF) that are expressed by CMs and cardiac fibroblasts with VEGF-B being the predominant growth factor in the heart (Zhang P. et al., 2012). Crosstalk between CM and non-CM is bidirectional, and disturbances in cell-cell communication has been implicated in the pathophysiology of, e.g., heart failure and arrhythmias (Lim et al., 2015).

When designing MPS, cell source and origin, as well as developmental and activation state of not only CMs but also other cell types should be considered. Ensuring consistent developmental stages of CMs and supporting non-myocytes may be a critical factor for promoting functional maturation of engineered cardiac tissues (Liau et al., 2017). Ideally, fibroblasts and EC of cardiac origin should be used due to site-specific differences in gene expression (Lim et al., 2015). Giacomelli et al. (2020) produced multicellular cardiac microtissues composed of hiPSC-derived CMs, cardiac fibroblasts, and cardiac ECs. By replacing primary ECs and fibroblasts with hiPSC counterparts, they were able to increase the microtissue throughput and, moreover, improve hiPSC-CM maturation (Giacomelli et al., 2020).

Relevant cell ratios and their spatial distribution are also important considerations for the design of cardiac MPS. In the diseased heart, relative cell proportions and cellular arrangement (Kanzaki et al., 2012) can change, e.g., due to fibroblast activation, CM apoptosis or immune cells invasion (reviewed in (Weber et al., 2013). Finally, cells maintain their phenotype in early passages but dedifferentiation should be carefully monitored in long term cell culture.

### 4.1 Endothelial cells

Endothelial cells (ECs) form the lining of blood vessels and a specific barrier structure between surrounding tissue and blood. ECs respond to blood flow derived shear stress changes through release of vasodilating and vasoconstricting factors such as nitric oxide (NO) or endothelin to communicate with surrounding stromal cells, such as smooth muscle cells and fibroblasts, and to adjust vessel tone (Chatterjee and Fisher, 2014). ECs are known to differ among tissues and even within the same tissue, such as heart, ECs comprise a heterogenous cell population due to differing regional needs and vessel size (Zhang et al., 2005; Litviňuková et al., 2020). In MPS development, tissue-specific ECs should be preferred, and mechanical forces implemented in the modeling platform to replicate the vascular *in vivo* environment more closely.

ECs of the coronary vessels form vascular network throughout the heart myocardium and have a different origin compared to ECs of the outer endocardium (Mikawa and Fischman, 1992). Already from early stage in development, interaction between CMs and ECs is necessary for proper growth and development of heart. Communication between CMs and ECs is processed through gap junction proteins, such as connexin 43 (Cx43), that links electromechanical processes in the myocardium with the surrounding vasculature (Givvimani et al., 2011). The main growth factor that regulates ECs is VEGF that binds to and activates VEGF receptor 2 in ECs (Olsson et al., 2006). VEGF mediated signaling induces angiogenesis, the growth of new blood vessels from the pre-existing ones, e.g., in ischemic areas or in a growing heart (Olsson et al., 2006).

In cardiac MPS, the benefit in integrating ECs can be detected with increased secretion of angiogenic growth factors and improved vessel forming capacity. Narmoneva et al. (2004) were the first ones to show the importance of EC-CM interactions in 3D environment *in vitro*. Their results demonstrated increased survival and spatial reorganization of CMs in the co-culture mediated partly via Cx43 gap junctions. The results of this study suggested an extended role of cardiac endothelium not only in the delivery of blood and oxygen but also in the formation and maintenance of structure in myocardium (Narmoneva et al., 2004). Sekine et al. (2008) showed that cardiac cell sheets with ECs secrete significantly more VEGF, bFGF, and hepatocyte growth factors (HGF) in comparison to the cardiac sheets without ECs. They also studied co-culture of fibroblasts and ECs and did not detect increased secretion of any of the angiogenic factors suggesting a specific interaction between CMs and ECs (Sekine et al., 2008). Endothelial-cardiomyocyte co-cultures have been established also in MPS where ECs are typically seeded to coat microfluidic channels in order to mimic blood flow (Ellis et al., 2017). In MPS, ECs experience perfusion derived shear stress, align according to the fluidic flow (Ellis et al., 2017). Importantly, they can be used to establish intra- and extra-vascular interactions with the surrounding cells and ECM with increased *in vivo* vascular resemblance compared to static cultures.

### 4.2 Stromal cells

Beside ECs, fibroblasts are the other major cell type present throughout the myocardium and interacting with CMs (Brutsaert, 2003). In the heart, fibroblasts are the primary cell type responsible for synthesis and degradation of ECM, and in providing mechanical support for the contracting CMs (Baxter et al., 2008). Additionally, cardiac fibroblasts play a crucial role in wound healing and repair, produce growth factors and other signaling molecules that directly regulate CM function (Brutsaert, 2003; Baxter et al., 2008; Ieda et al., 2009; Howard and Baudino, 2014).

In MPS design, developmental stage of the used fibroblasts should be considered since, e.g., embryonic fibroblasts have been shown to promote proliferation of CMs whereas adult fibroblasts promote CM hypertrophy, i.e., increased size of CM (Ieda et al., 2009). After cardiac tissue injury, due to, e.g., myocardial infarction, fibroblasts are the main cell type contributing to fibrotic scar formation through ECM production. Fibroblasts from the infarcted region present phenotype with increased proliferative and collagen synthesis capacity (Squires et al., 2005). In development of MPS disease models, fibroblasts should be considered as an essential cell type for mimicking the *in vivo* situation of cardiac injury.

Although cardiac fibroblasts are considered as non-excitable cells, they have been shown to modulate electrical properties of CMs. Kofron et al. (2017) showed that cardiac fibroblast activation altered AP and Ca^2+^ transients of CMs and lead to proarrhythmic activity of CMs (Kofron et al., 2017). Through gap junction protein Cx43 and Cx45 connections, fibroblasts and CMs are able to communicate with one another electrically. Furthermore, by fibroblast coupling, fibroblasts may form bridges and allow CMs in different cardiac locations to communicate with one another (Kamkin et al., 2005).

Several studies have demonstrated that fibroblasts improve hiPSC-CM viability and structural and functional maturity (Baudino et al., 2008; Tiburcy et al., 2017; Giacomelli et al., 2020) in 3D environment that more closely mimics the native myocardium (Beauchamp et al., 2020; de Lange et al., 2021). hiPSC-CMs in 3D microtissues with cardiac fibroblasts showed improved sarcomeric structures with T-tubules, enhanced contractility, and mitochondrial respiration and were electrophysiologically more mature compared to microtissues lacking fibroblasts (Giacomelli et al., 2020). In addition to fibroblasts, human mesenchymal stem/stromal cells (MSCs) have been shown to improve hiPSC-CM structural maturation, contractility and electrophysiological development when co-cultured *in vitro* or co-transplanted *in vivo* (Yoshida et al., 2018). Bone marrow-derived MSCs secreted soluble factors, including VEGF, bFGF, stromal cell-derived factor 1, granulocyte-macrophage colony-stimulating factor growth factors and exosomes, enhanced hiPSC-CM mitochondrial energetics, performance and reduced reactive oxygen species production by CMs under stress (Yoshida et al., 2018).

Beside improving CM maturation, fibroblast have been shown to support formation of vascular network in 2D (Vuorenpää et al., 2014) and in 3D cardiac *in vitro* models (Caspi et al., 2007). Similar contribution to angiogenesis has been detected with MSCs when compared to EC monocultures in vascularization studies (Sato et al., 2011; Sarkanen et al., 2012; Jeon et al., 2014; Mykuliak et al., 2022). In the next chapter, we will review the current methods to vascularize cardiac *in vitro* models.

### 4.3 Vascularization in cardiac *in vitro* models

It is estimated that humans have approximately 100,000 km of blood vessels networking throughout the body thus making vascular endothelium the largest endocrine organ (Talman and Kivelä, 2018). Blood vessels support heart with oxygen, nutrients and signaling molecules and interact with the surrounding tissue to maintain homeostasis. The continuous activity of heart creates a large demand for oxygen and nutrient delivery to cardiac tissue and, therefore, one capillary resides next to every cardiomyocyte in normal adult myocardium (Brutsaert, 2003). Function as selective barrier and as an interface between blood and the surrounding tissue is critical in several (patho-)physiological processes (Talman and Kivelä, 2018; Browne et al., 2021).

In EHTs, hydrogel mass transport properties can be adjusted to replace vascular functions, but only to a certain limit. The mesh size of hydrogels is typically in the nanometer scale and pore size in the micrometer scale (Li and Mooney, 2016). Both affect how molecules are allowed to move inside these water filled regions in the scaffolds (Karvinen et al., 2019). In addition, the solute molecules may be bound by electrical charges on the polymer chains (Amsden, 1998). Diffusion properties and the need for vascularization becomes important when the diffusion distances (Soto et al., 2016) and cell densities increase (Figueiredo et al., 2018).

Vascularization has been one of the main targets in development of MPS and can be considered in two level; vascular network formation inside individual tissue model and circulation between tissue models to establish connected MPS. Both means of vascularization are essential in mimicking tissue level functions, and in increasing physiological relevance and accuracy of MPS. Vascularization inside tissue model (1) enables to overcome the growth limit (∼200 µm) inside a tissue model dependent on diffusion of oxygen and nutrients (Browne et al., 2021); (2) improves target cell differentiation and maturation (Caspi et al., 2007; Vuorenpää et al., 2014; Vuorenpää et al., 2017) and (3) enables 3D structure, mechanical support and growth factor related stimulation for other cell types (Sarkanen et al., 2012; Vuorenpää et al., 2014). Connective circulation between tissue models is important in delivering nutrients, oxygen, signaling molecules and test substances such as drugs between tissue models. It enables (1) long term viability of the cells; (2) metabolic activities; (3) tissue-tissue signaling; (4) implementation of mechanical forces such as shear stress and interstitial flow, and, importantly, (5) environmental control.

There are several methods to vascularize MPS (Figure 3B). ECs can be cultured on designed patterns or engineered interfaces using biofabrication techniques such as sacrificial molds (Paek et al., 2019; Kim et al., 2021) or tunnels made with laser-based cavitation molding (Enrico et al., 2022). Additionally, ECs are used to coat or endothelialize surfaces (Paek et al., 2019; Zhang et al., 2020) or induced to self-assemble into microvasculature (Kim et al., 2013; Jeon et al., 2014; Mykuliak et al., 2022). Ability to form perfusable vascular structures with intact vessel wall and open lumen increases the physiological relevance of the vasculature and enables, therefore, more complex applications such as immune cell extravasation studies (Kim et al., 2012; Gopalakrishnan et al., 2015; Wu et al., 2015; Marzagalli et al., 2022). In establishing perfusable vascular model in reproducible manner, EC source and passage are shown to be critical. We and others have shown (Liu Y. et al., 2020; Mykuliak et al., 2022) that vasculogenesis, *de novo* production of blood vessels, is critically impaired when EC passaging exceeds 7–8. Moreover, vasculogenesis process leading to perfusable microvasculature is not dependent only on the culture time but also on the cell source. Human umbilical vein endothelial cells are still considered as a gold standard in vascularization due their robustness, easy availability andfor their ability to adapt in different tissue engineering applications (Vuorenpää et al., 2014; Liu Y. et al., 2020; Calejo et al., 2020). However, iPSCs provide an option to receive not only personalized or patient-specific ECs but also ECs with tissue specific characteristics for MPS development (Browne et al., 2021).

Although EC monocultures form vascular structures with lumen *in vitro,* supporting pericytes significantly contribute to angiogenesis and improve vascular maturation (Sarkanen et al., 2012; Jeon et al., 2014; Liu Y. et al., 2020; Mykuliak et al., 2022). The importance of pericytes in physiological blood vessel formation has been shown in several cardiac applications (Caspi et al., 2007; Vuorenpää et al., 2014; Vuorenpää et al., 2017) and, more recently, in pathological conditions such as cardiac ischemia (O’Farrell et al., 2017) and atherosclerosis (Zhang et al., 2020). Different types of supporting cells such as fibroblasts, smooth muscle cells and MSCs have been used in co-culture with ECs in MPS (Jeon et al., 2014; Fernandez et al., 2016; Mykuliak et al., 2022) and in cardiac *in vitro* models (Caspi et al., 2007; Vuorenpää et al., 2014; Vuorenpää et al., 2017). Caspi et al. (2007) were the first ones to show the generation of vascularized 3D human cardiac *in vitro* model based on co-culture of hESC-CM, EC and fibroblasts. Importantly, they showed that vascularization resulted in upregulation of early and late markers of CM differentiation and maturation (Caspi et al., 2007). Also in regenerative medicine, the benefit in pre-vascularizing cardiac tissues instead of transplanting single CMs has been widely acknowledged (Sekine et al., 2008; Stevens et al., 2009; Masumoto et al., 2016; Ishigami et al., 2018).

Earlier, we reported 2D cardiac construct with ECs, fibroblasts and hiPSC-derived CMs showing aligned vascular network with CMs and an increased expression of cardiac structural proteins compared to CM monoculture (Vuorenpää et al., 2017). Also an elegant study by King et al. (2022) highlights the role of non-CMs as fundamental regulators of iPSC-CM electrophysiology by demonstrating that regulation of beating rate and Ca^2+^ transients were lost when cells were physically separated. In heart-on-a-chip, they were able to show establishment of perfusable lumen with intra-vascular red blood cell transport and pulsatile red blood cell velocity according to CM contractile cycle (King et al., 2022). For MPS development in general, similar physiological vascular flow utilizing CM contractile function as pump mechanism may be beneficial.

### 4.4 Innervation

Tissues are mostly consisted of other cell types than neuronal cells. Still, their important functions and behavior patterns are controlled via nerve stimulation (Park et al., 2020). In addition to CM, fibroblasts and endothelial cells, also network of neuronal cells is important part of the human heart (Figure 3A) (Zaglia and Mongillo, 2017). The heart is a densely innervated organ. The high number of neurons in the heart has been recognized, but extent of the innervation density has been underestimated (Zaglia and Mongillo, 2017). In general, the innervation has crucial role in tissue development as well as in their functional control, modulation, and regeneration and therefore would be important part to consider including in the MPSs. Despite its importance, innervation for the functionality of the tissue has been generally overlooked in many non-neuronal applications (Das et al., 2020). Only a few cell types have been studied *in vitro* with integrated innervation, such as mesenchymal stem cells, muscle cells, retinal pigment epithelial cells, human adipose stem cell derived corneal keratocytes, and heart cells (Morimoto et al., 2013; Silva et al., 2017; Yeste et al., 2017; Mörö et al., 2022).

Cardiac nervous system (NS) innervate myocardium and cardiac conduction system (Habecker et al., 2016). It is comprised of sympathetic, parasympathetic and sensory neurons, and is regulated by adrenergic and cholinergic signaling via neuromodulator molecules norepinephrine and acetylcholine (Elia and Fossati, 2023). All cardiac neurons are originally developed from neural crest cells (Tampakakis and Mahmoud, 2021) and each of these neuronal cell populations has certain phenotype and distribution which is crucial for their functions (Habecker et al., 2016). In humans, the cell bodies of the cardiac preganglionic neurons are located in grey matter of the thoracic spinal cord. Only few postganglionic neuron cell bodies have been found in the myocardium (Coote and Chauhan, 2016).

The cardiac functions are regulated by sympathetic and parasympathetic neurons. During development, sympathetic neurons and CMs undergo co-maturation process meaning that signals from cardiac tissue regulate properties such as growth and patterning of the sympathetic neurons whereas maturation of the CMs is influenced by signaling of the nerves (Habecker et al., 2016). In addition, sympathetic neurons play important role in postnatal maturation of the CMs (Tampakakis and Mahmoud, 2021). The sympathetic neurons that predominantly innervate heart are located in stellate ganglion adjacent to the spinal cord segments T1-T4. When they enter through epicardium to the heart they extend and branch out to the myocardium (Odnoshivkina and Petrov, 2021). The sympathetic nervous system has important role in maintaining homeostasis in healthy and diseased state of the heart. The main role of sympathetic neurons is to increase heart rate, cardiac contractility and conduction velocity as well as regulate blood pressure. Parasympathetic neurons act in an opposite manner, they decrease heart rate, contractility and conduction velocity (Das et al., 2020). Sensory nerves are responsible for pain perception and starting protective cardiovascular response in ischemia (Kimura et al., 2012).

Unlike neuromuscular junctions, synaptic junctions between neurons and cardiac tissue are not that well understood. It has been shown that protein complexes are involved in cellular interactions in case of sympathetic neurons and CMs or in exocytosis. These protein complexes include post-synaptic β-adenoreceptors in CM cell membrane (Shcherbakova et al., 2007). However, it is known that crosstalk between the sympathetic neurons and CMs occur via contact of varicosities, clusters of extensions filled with neurotransmitters located in the nerve endings, and CMs (Odnoshivkina and Petrov, 2021).The dysregulation and abnormalities in cardiac innervation cause impairment in the cardiac functionality and cardiac nerve fiber abnormalities, and are thus linked to the life threatening arrhythmias, congestive heart failure and myocardial infarction (Bairey Merz et al., 2015; Elia and Fossati, 2023). In diseased state, change in oxidative stress and expression of biomolecules (growth factors and cytokines) in heart and vasculature leads to changes in phenotype and morphology in cardiac nerves (Habecker et al., 2016).

Examples of the previously reported methods to innervate cardiomyocytes are collected to the Table 1. Major of the applications are still conducted in 2D platforms, either as simple co-culture of mixed cells (Winbo et al., 2020) or in microfluidic devices which support axon-mediated innervation (Takeuchi et al., 2011; Takeuchi et al., 2013; Oiwa et al., 2016; Takayama and Kida, 2016; Sakai et al., 2017; Häkli et al., 2022a; Bernardin et al., 2022). There are still only a few reports describing cardiac innervation performed in 3D environment (Soucy et al., 2020).

**TABLE 1:** Summary of innervation applications of the cardiomyocytes.

Application	Features	CM	Neurons	Platform	2D/3D	References
Co-culture using microfabrication	Electrical stimulation and measurement with MEA	Rat ventricular CM	Rat cervical ganglion neurons	Compartmentalized microfluidic chip	2D	Takeuchi et al. (2011)
Co-culture using microfabrication	Electrical stimulation and measurement with MEA	hiPSC-CM	Rat cervical ganglion neurons	Compartmentalized microfluidic chip	2D	Takeuchi et al. (2013)
Effect of sympathetic and parasympathetic neurons to cardiomyocytes in co-culture	Electrical stimulation and measurement with MEA	Rat atrial myocytes	Rat sympathetic neurons and rat parasympathetic neurons	Compartmentalized microfluidic chip	2D	Oiwa et al. (2016)
Effect of sympathetic neurons to hiPSC-CM (MEA)	Electrical stimulation and measurement with MEA	hiPSC-CM	Rat sypathetic neurons	Compartmentalized microfluidic chip	2D	Sakai et al. (2017)
Functional co-culture	-	hiPSC-CM	hiPSC-peripheral neurons	Compartmentalized microfluidic chip	2D	(Takayama et al., 2020)
Cardiac sympathetic innervation and multitissue interactions	Possible to integrate with commercial MEA	Rat primary CM	Rat primary postganglionic sympathetic neurons	Compartmentalized microfluidic chip	3D	Soucy et al. (2020)
Functional co-culture on coverslip	Functionality with patch clamp	hiPSC-CM	hiPSC-sympathetic neurons	12 mm coverslips	2D	Winbo et al. (2020)
Functional connections between CM and neurons	Functional analysis and chemical stimulation with video-based analysis	hiPSC-CM	hiPSC neurons	Compartmentalized microfluidic chip	2D	Häkli et al. (2022a)
Neuro-cardiac junctions	Functionality with calcium imaging and video-based analysis	hiPSC-CM	Rat sympathetic neurons and hiPSC autonomic neurons	Compartmentalized microfluidic chip	2D	Bernardin et al. (2022)

CM, Cardiomyocyte; hiPSC, human induced pluripotent stem cell; MEA, Microelectrode array.

The significant benefit of the microfluidic devices for innervation applications is the restriction of the cell somas from the target cells or tissues with compartmentalized structures and microtunnels (Figure 3B). These structures allow assessment of the axon-mediated innervation and mimics *in vivo* conditions of central nervous system and peripheral nervous system better (Neto et al., 2016; Park et al., 2020; Pelkonen et al., 2020; Ristola et al., 2021; Tong et al., 2021; Häkli et al., 2022a). In addition, *in vitro* neuronal network models have shown to share many similarities with *in vivo* networks. This aspect increases the relevance of the model for preclinical tests and drug screening.

Currently, the importance of innervation for the functionality and development of other tissue structures has still mostly been shown in co-cultures with only one cell type, or in simple multicellular cultures, not yet in more complex MPS systems. While studies combining CMs and neuronal cells are already concentrating on investigating physiological conditions with hiPSC-CMs, still mostly animal derived neuronal cells are used. However, completely human based models are needed for neuro-cardiac research for better translation of human physiology. Current trend goes towards more clinically relevant cell types, better *in vivo* conditions mimicking *in vitro* models and MPS and towards 3D environment in the future.

## 5 Physical stimulation and supporting matrix

The focus of this part is to offer a review on iPSC-CM response to physical stimulation, namely, mechanical stimulation, including the physical properties of the surrounding matrix, and electrical stimulation. There are already excellent reviews on mechanical and electrical stimulation in cardiac applications (Carlos-Oliveira et al., 2021; Dai et al., 2021; Cho et al., 2022). Instead, here we aim to summarize the effect of physical stimulation on hiPSC-CM maturation. Optogenetic stimulation has also raised interest as maturation tool (Dwenger et al., 2021) but to our knowledge, it is currently only used on modulating tissue excitability by pacing (Gruber et al., 2022). It will therefore be excluded from the scope of this chapter.

**FIGURE 4:**
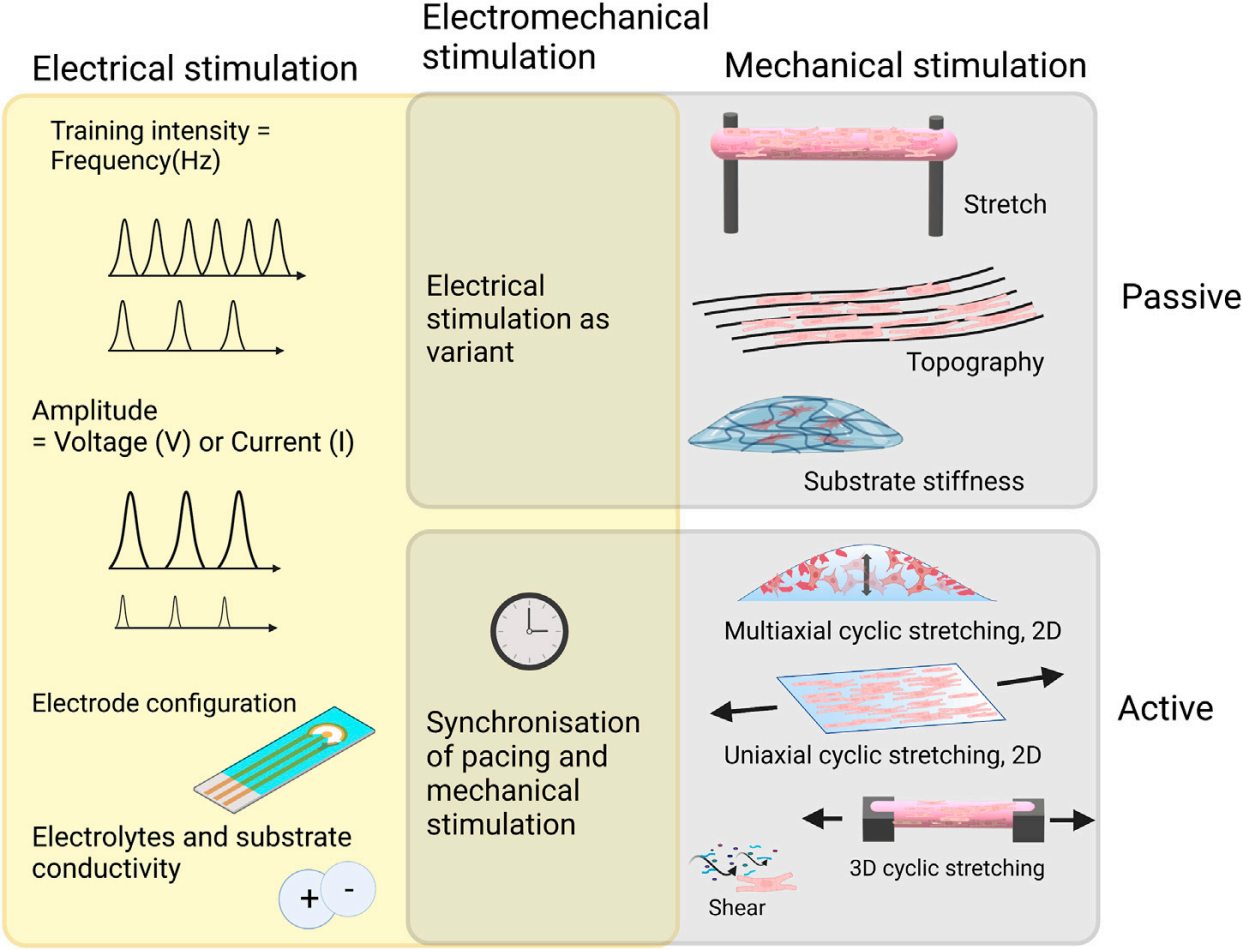
Commonly used mechanical stimulation methods for cardiac maturation of hiPSC-CM and important considerations when implementing electrical stimulation with or without mechanical stimulation. Created with BioRender.com.

*In vitro* physical stimulation is used for mimicking several phenomena in the heart during different developmental stages. Mechanical cues can be used for mimicking 1) shear stress caused by blood flow and followed by vascular tube formation, 2) cyclic strain manifesting the systolic and diastolic rhythm from the beginning of the first heartbeat, 3) hydrostatic stretching due to increased blood pressure and 4) increase in the elastic modulus of ECM from birth to adulthood as previously summarized in the reviews of (Hendrickson et al., 2021) and (Carlos-Oliveira et al., 2021). Mechanical stimulation can be categorized into passive and active stimulation. Passive stimulation can relate to the properties of the surrounding matrix (stiffness and topography that the cells experience) or passive stretch in which cells or cell construct is fixed on passive anchors (Gaetani et al., 2020; Guo and Pu, 2020). In active mechanical stimulation, external changing forces are directed to the cells for a stretching or compression purpose (Kreutzer et al., 2020; Peussa et al., 2022). A schematic of commonly used physical stimulation methods for cardiac maturation of hiPSC-CM is presented in Figure 4.

*In vitro* electrical stimulation draws the analogue from the inherently electrical environment of cardiac tissue. Sinoatrial node releases rhythmic electrical impulses via purkinje fibers causing rhythmic contraction of cardiomyocytes. *In vitro* electrical stimulation can be used for maturation of the hiPSC-CM, which is further reviewed in this chapter. Other purposes for using electrical stimulation include functional analysis to study contraction dynamics and calcium transients (CTs; transient change in intracellular Ca^2+^ concentration) which are introduced in chapter 6.

### 5.1 Supporting matrix

2D cell culture methods do not allow the cells to be in natural contact with other cells and the ECM. For better biomimicking, cell cultures adding a spatial dimension is necessary. Different 3D culturing approaches have shown success with hiPSC-CMs. It has been previously demonstrated already with 2D island cultures that dimensionality affects hiPSC-CM maturation (Werley et al., 2017). In comparison to a planar culture, a 3D culture allows even more of cell-cell and cell-matrix contact. To this date many hiPSC-CM 3D cell cultures have been developed (Andrysiak et al., 2021).

Substrate stiffness and topography can greatly affect hiPSC-CM maturation. Soft substrates close to the physiological range of myocardium are often reported to promote structural and functional maturation in comparison to rigid surfaces (Körner et al., 2021; Pioner et al., 2022). However, the concept of the substrate stiffness is complex and cell response greatly depends on spatial dimension (e.g., 2D vs. 3D) and topography, such as aligned grooves, and the choice of material. To add complexity, myocardium is anisotrophic and stiffness values vary greatly between longitudinal and cross sectional parameters, which makes mimicking of the physical properties of the native matrix challenging (Querceto et al., 2022). The effects of substrate stiffness and different materials on hiPSCs have been summarized in Table 2.

**TABLE 2:** Hydrogel scaffold stiffness in hiPSC-CM cell cultures.

Hydrogels	Stiffness	Cells	2D/3D	References
Collagen	22 kPa, glass	hiPSC-CMs	2D	Huethorst et al. (2022)
PEG-DA, DEG-DA	not specified	hiPSC-CMs	2D patterns	Pioner et al. (2022)
PDMS	5–30 kPa	hiPSC-CMs	2D	(Guo et al., 2021)
PAAm-co-PAAc	0.56–112 kPa	hiPSC-CMs	2D	Kit-Anan et al. (2021)
dECM-rGO	10.3–18.0 kPa	hiPSC-CMs, human bone marrow-derived stromal cells	3D	Tsui et al. (2021)
Fibrin, Gelatin	0.6–9.2 kPa	hiPSC-CMs, human cardiac fibroblasts	2D and 3D	Mastikhina et al. (2020)
Polyacrylamide	4–101 kPa	hiPSC-CMs	2D patterns	Ribeiro et al. (2020)
Gelatin-Gellan gum	23–300 kPa	hiPSC-CMs, human lung fibroblasts	2D and 3D	Koivisto et al. (2019)
PEG-DA	100 kPa	hiPSC-CMs	2D patterns	Pioner et al. (2019)
Polyacrylamide	3.1–13.5 kPa	hiPSC-CMs, neonatal rat CMs	2D patterns	Wheelwright et al. (2018)
Polyacrylamide	4–16 kPa, glass	hiPSC-CMs	2D	Martewicz et al. (2017)
Gelatin	2–16 kPa	hiPSC-CMs	3D	Lee et al. (2017)
Matrigel	5.8 kPa	hiPSC-CMs, adult rabbit ventricular CMs	2D	Feaster et al. (2015)
Matrigel	6–35 kPa	hiPSC-CMs	2D patterns	Ribeiro et al. (2015)
Polyacrylamide	4.4–99.7 kPa	hiPSC-CMs, MEFs, neonatal rat CMs	2D	Hazeltine et al. (2012)

CM, Cardiomyocyte; hiPSC, human induced pluripotent stem cell.

In 2D configuration hiPSCs have been cultured on substrates ranging from soft hydrogel (0.56–300 kPa) and PDMS (5–30 kPa) to rigid glass (25 GPa) as shown in Table 3. Rigid (>1 GPa) surfaces were found to cause unphysiological contractions for hiPSC-CMs meanwhile soft (22 kPa) hydrogel substrate allowed normal contractility (Huethorst et al., 2022). In a single cell study, where hiPSCs were compared between stiffer (112 kPa) 2D surfaces and softer (9.83 kPa) 3D configuration, cells exhibited increased Cx-43 density, cell membrane stiffness and CT amplitude in the latter (Kit-Anan et al., 2021).

**TABLE 3:** Selected studies of the effect of mechanical stimulation on hiPSC-CM maturation. Experimental groups of the studies are renumbered for easier interpretation of this table and may not be labelled similarly in the original articles.

Type of stimulation	Parameters	Effect of physical stimulus on hiPSC-CM maturation	References
Passive mechanical stimulation of EHT	• 12.5% uniaxial stretch	• Growth, regulation of sarcomere and extracellular matrix synthesis and organization↑	Lui et al. (2021)
		• Flexibility and beat contractile strength	
		• Expression of cardiac cell identity markers ↑	
	• Afterload forces between 0.09 up to 9.2 μN/μm	• mid-range afterload: sarcomere length, cardiomyocyte area, elongation ↑	Leonard et al. (2018)
		• Progressive afterload: a shift from fetal to adult ventricular myosin heavy chain isoforms	
Active mechanical stimulation	• Prestretching: 0.5 Hz; 1.5% for 1 min, 3% for 2 min and 8% for 7 min	• Organization and sarcomere orientation of the cells perpendicular to uniaxial strain axis	Kreutzer et al. (2020)
	• Cyclic stress of 8% elongation, 0.8 Hz for 2, 4 or 7 days		
	• 1 Hz cyclic stretching to generate a 10% strain for 48 h	• Expression of genes related to nuclear mechanotransduction, actin alignment, sarcomere length in the direction of the stretch. ↑	Song et al. (2022)
	• Strains of 10% (0.5 Hz, sine wave), stepwise increase from day 0%–10% strain after 8 h	• No effect when applied parallel to the anisotropic ECM.	Mostert et al. (2022)
		• Isotropic substrates, CMs show strain avoidance via active remodeling of their sarcomeres only when co-cultured with at least 30% cardiac fibroblasts	
Electrical or active mechanical stimulation (comparison)	• Group 1 (aMS): 10% strain at a frequency of 1 Hz	• Group 1–2: transcript and protein expressions for key maturation markers, ultrastructure, Z-band/Z-body formation, fibril alignment, and fibre number ↑	LaBarge et al. (2019)
	• Group 2 (ES): 6.5 V/cm with 5 ms pulses, 2 Hz		
Active electromechanical (only mechanical studied)	• Group 0 (aMS): 0 mm/day	• Group 3: force development ↑ by 5.1-fold. Positive force-frequency relationship	Lu et al. (2021)
	• Group 1 (aMS): 0.08 mm/day, Group 2 (aMS): 0.16 mm/day, Group 3 (aMS): 0.32 mm/day)	• Group 1–3: cellular volume, linear alignment, and sarcomere length, adult cardiomyocyte genes ↑	
	• ES: 1 Hz and 50 mA current		

ES, Electrical stimulation; pMS, passive mechanical stimulation; aMS, active mechanical stimulation; EM, electromechanical stimulation.

In 3D, hiPSC-CM have shown stronger maturation characteristics in 9 kPA and 2 kPA gelatin hydrogel in comparison to 16 kPa hydrogel (Lee et al., 2017). Martewicz et al. reported that RhoA/ROCK mechanotransduction pathway is involved in hiPSC-CMs sensing the stiffness of the material (Martewicz et al., 2017). Ribeiro et al. reported that the material stiffness affects hiPSC-CM sarcomere dynamics up to the physiological levels whereas stiffness values beyond that did not seem to have an increasing effect (Ribeiro et al., 2020).

Inhomogeneities within the hydrogel scaffolds after preparation can cause variations in the stiffness each cell is experiencing (Soto et al., 2016). The stiffness also changes over time as the cells remodel their environment as is shown with microrheological measurements (Hafner et al., 2020; Koivisto et al., 2022). It is important to acknowledge that the cells in one culture are sensing varying stiffnesses. Measuring the stiffness of the whole bulk scaffold gives useful insight into the mechanical properties, while characterizing the cells’ actual microenvironment does give more realistic insight to the situation.

### 5.2 Passive stretch

Passive stretch in 3D EHTs is done by fixing the constructs between posts. The distance of the posts is either kept the same throughout the study or increased in stepwise manner mimicking the dynamic changes in the myocardium during development. While stretch creates tension in the cell structures, it also orientates the cells in the direction of stretch (Ruan et al., 2016; Lui et al., 2021). Leonard et al. tested different increases of afterload on hiPSC-CM-based EHT (Leonard et al., 2018). Afterload is the systolic load on the left ventricle during a contraction (Kligfield, 1998). From afterload forces between 0.09 up to 9.2 μN/μm the mid-range was discovered to most improve the maturation of hiPSC-CM in terms of increased sarcomere length, cardiomyocyte area and elongation. Progressive afterload was found to increase several key markers of maturation including a shift from fetal to adult ventricular myosin heavy chain isoforms. Interestingly, markers of pathological hypertrophy and fibrosis were upregulated at the highest afterload condition (Leonard et al., 2018). The effect of mechanical stimulation on hiPSC maturation is summarized in Table 3.

### 5.2 Active mechanical stimulation

In active mechanical stimulation, cells are stimulated externally with changing forces rather than causing the stimulus, for example, by the contracting cells attached to fixed anchors. In comparison to passive mechanical stimulation, significantly less studies are done on the active mechanical stimulation of hiPSC-CM. Song et al. studied the effect of 1 Hz cyclic stretching to generate a 10% strain for 48 h on hiPSC-CM in 2D PDMS platform. Cyclic stretching increased the expression of genes related to nuclear mechanotransduction. Actin alignment and sarcomere length was higher in the direction of the stretch. Beating rate was similar in both groups (Song et al., 2022). In contrast, in our previous 2D study with cyclic stretching (0.5 Hz pre-stretch with 8% elongation following 0.8 Hz stretch with 8% elongation) of hiPSC-CM on PDMS substrate showed orientation of cells perpendicular to the direction of stretch (Kreutzer et al., 2020). Different magnitudes of 1 Hz cyclic stretching were compared by Dou et al. using building 2D PDMS membrane as the substrate for hiPSC-CM (Dou et al., 2022). The contractile stress of the cells increased with increased strain (0%, 5%, 10%, 15% and 20%) up to 15% of strain, where it plateaued. Strain of 20% did not increase contractile stress. Mechanical stress overall increased intercellular alignment and sarcomere orientation to radial direction (circumferential) and MYH7 protein expression.

Shear stress for mimicking the early stage of heart tube development has been studied for hiPSC-CM in an MPS system by Kolanowski et al. (Kolanowski et al., 2020). Pulsatile fluid flow generated in the MPS system increased several indicators of maturation such as the beating intensity, sarcomere length, mitochondrial density, and the elongation of the mitochondrial network.

### 5.3 Electrical stimulation with or without passive mechanical stimulation

Electrical stimulation for maturation of hiPSC-CM has been more widely studied than active mechanical stimulation. The reason for this could be that electrical stimulation is relatively easy to implement in comparison to active mechanical stimulation where the accurate anchoring of cells could pose additional challenges. This chapter encompasses also those studies made on hiPSC-CM that include mechanical stimulation with electrical stimulation but have not compared those conditions to mechanically non-stimulated controls. Therefore, they focus solely on studying the effect of electrical stimulation. The effect of electrical stimulation on hiPSC maturation is summarized in Table 4.

**TABLE 4:** Selected studies of the effect of electrical and electromechanical stimulation on hiPSC-CM maturation. Experimental groups of the studies are renumbered for easier interpretation of this table and may not be labelled similarly in the original articles.

Type of stimulation	Parameters	Effect of physical stimulus on hiPSC-CM maturation	References
Electrical stimulation of biowires (hiPSC-CM, fibroblasts, endothelial cells and smooth muscle cells)	• Group 1: ES, Low-frequency ramp-up regimen	• Increased myofibril ultrastructural organization	Nunes et al. (2013)
	• Group 2: ES, high-frequency ramp-up regimen	• Elevated conduction velocity	
		• Improved both electrophysiological and Ca^2+^ handling	
Electrical stimulation of human EHT	• ES: Biphasic 2 Hz stimulation in the first week and 1.5 Hz thereafter	• 1.5 × higher contraction forces	Hirt et al. (2014)
Electrical stimulation of embryoid bodies (EBs of hiPSC-CM)	• ES: 1 V/cm or 1.5 V/cm square pulse (5 ms) at 5 Hz frequency for 1–30 days	• EBs with electrical stimulation started spontaneously contracting at 2.1 ± 0.40 days (controls at 7.0 ± 0.63 days)	Ma et al. (2018)
		• Expression of structural genes ↑	
		• Ca^2+^/PKC/ERK pathways	
Passive electromechanical stimulation	• Control: no stress	• Group 1: tensile stiffness, construct alignment, and cell size ↑	Ruan et al. (2016)
	• Group 1 (pMS): 2 weeks of static stress	• Group 2: additional increase in force production in comparison to group 1	
	• Group 2 (pEM): After 1-week of pMS, combined pMS and ES with 2 Hz, 5 V/cm, 5 ms pulse		
	• Control (pMS)	• Early stage hiPSC-CM in group 2 displayed unprecedented adult-like maturation markers than late stage hiPSC-CM.	Ronaldson-Bouchard et al. (2019)
	• Group 1 (pEM): Constant, 3 weeks at 2 Hz		
	• Group 2 (pEM): Intensity training (2 weeks at a frequency increasing from 2 to 6 Hz followed by 1 week at 2 Hz		
	• Control: no stimulus	• Group 2: alignment of contractile proteins, biomolecular maturation	Pretorius et al. (2022)
	• Group 1 (pMS): Stretched		
	• Group 2 (pEM): pMS and low-voltage (15V)		
	• Group 3 (pEM): pMS and high-voltage (22 V)	• Groups 1–3: development of Z-lines and gap junctions ↑ and sarcomere length ↑	
	• ES: 2-ms pulses at a frequency of at 2 Hz (Groups 1 and 2)		
Active electromechanical stimulation	• Group 1 (aMS): Uniaxial cyclic stretch 5%, 50% duty cycle with 16% expansion, 18% hold phase, 16% contraction to original length	• Group 1–3: sarcomere length was the shortest in all stimulated groups. No alteration in the structural phenotype	Kroll et al. (2017)
	• Group 2 (ES): ES: biphasic pulses of 3 V/cm voltage and 4 ms duration	• Group 3: myofibrillogenesis ↑ transmembrane calcium current	
	• Group 3 (aEM)		

ES, Electrical stimulation; pMS, passive mechanical stimulation; aMS, active mechanical stimulation; EM, electromechanical stimulation.

By varying the pulse frequency of electrical stimulation, different “training regimes” can be applied for striated muscles. The state of maturity of hiPSC-CM has also shown to play a role when responding to electrical stimulation. Ronaldson-Bouchard et al. hypothesized that electrical stimulation should be initiated to early hiPSC-CM, which still have high developmental plasticity, because responsiveness to physical stimuli declines as differentiation progresses (Ronaldson-Bouchard et al., 2018). In addition, a gradual ramp-up of the frequency of electrical stimulation each day pushes hiPSC-CM to adapt on the workload (Ronaldson-Bouchard et al., 2018; Ronaldson-Bouchard et al., 2019). They compared constant 2 Hz monophasic (4.5 V/cm) stimulation for 3 weeks, with “high intensity training” parameters where the frequency increases 0.33 Hz each day up to 6 Hz by day 12 (Ronaldson-Bouchard et al., 2019). Because of the pillar configuration, passive mechanical stimulation was triggered by contractions of the tissue structures. However, the conditions were not compared to non-static conditions. The stage of maturation with the early-stage high intensity-trained hiPSC-CM was at similar levels as achieved with previous biowire configuration (Nunes et al., 2013). Only these hiPSC-CM displayed orderly signal propagation and anisotropic gap junctions and exhibited positive force-frequency relationship (Ronaldson-Bouchard et al., 2018).

The voltage amplitude is another major variant that needs to be balanced case-to-case depending on for instance electrode materials, distance between electrodes and the pulse width (Björninen et al., 2018). Pretorius et al. compared low voltage (15 V) and high voltage (22 V) stimulation on hiPSC-CM with the commonly used C-Pace (IonOptix) stimulator (Pretorius et al., 2022). Cell constructs were manufactured into triple-layered cardiomyocyte patches and 2 ms voltage pulses at 2 Hz was applied for the patches under static stretching. Patches with static stretching and with no stretching were used as controls. The stretched patches with 22 V stimulation showed greatest expression and alignment of contractile proteins. Stretching and electrical stimulation seemed to cause greater development in Z-lines and gap junctions. Sarcomeres were significantly longer in any of the stimulated group compared to the non-stretched control.

Ruan et al. compared static stretch and that added with electrical stimulation. They found that while static stretch of hiPSC-CM increased in contractility, tensile stiffness, construct alignment, and cell size, additional electrical stimulation increased force production. They suspected that electrical stimulation promoted maturation of excitation-contraction coupling as expression of RYR2 and SERCA2 increased (Ruan et al., 2016).

### 5.4 Active electromechanical stimulation

Active electromechanical stimulations (mechanical stimulation is active), where both the effects of mechanical stimulus and electrical stimulus are compared together or separately, are still few. Lu and coworkers applied progressive stretch on EHTs and compared with those subjected to static stress (Lu et al., 2021). Electrical stimulation parameters were kept constant. Posts, where EHTs were anchored were moved further from the other post daily and electrical stimulation was applied immediately after. The highest rate of stretch resulted in greatest force development. The role of electrical stimulation remained unclear as it was not compared to a control without electrical stimulation.

The limitless options of cell culture conditions and parameters in electromechanical stimulation makes the area particularly challenging to navigate. The stimulus, that ought to be mimicking physiological conditions, can also have a negative effect on maturation markers. Such effect is demonstrated by Kroll et al. who tested elecromechanical stimulus on hiPSC-CM up to 7 days of stimulation (Kroll et al., 2017). After each stretching phase of cyclic stretch of 5%, electrical stimulation was applied. Both stimulation types were also separately tested in separate groups. The electromechanical stimulus aimed to mimic the filling of the ventricles and an isovolumetric contraction. The cells had been synchronously beating for 3 days before the start of the stimulus. Neither of the stimulation regimes altered structural phenotype of the cells. Surprisingly sarcomere length was the shortest in all stimulated groups whereas non-stimulated control was nearly on the level of the optimal range in human heart tissue ranging in 1,9–2.2 μm (Hamdani et al., 2007). The greatest effect on myofibrillogenesis was observed with the EM stimulation which also significantly reduced the transmembrane calcium current. The authors speculated that a gradual increase in stimulation would be required to alleviate the stress response of the cells (Kroll et al., 2017).

## 6 Functional measurements

Heart cell responses to drug-induced and microenvironmental changes can be roughly divided into structural and functional responses (Arslan et al., 2022). While the structural responses can be examined with various microscopies, functional responses requires dedicated sensor technologies. These building blocks may contain various physical and (bio-)chemical sensors and analyzers working *in situ*, in-line, on-line or off-line.

**FIGURE 5:**
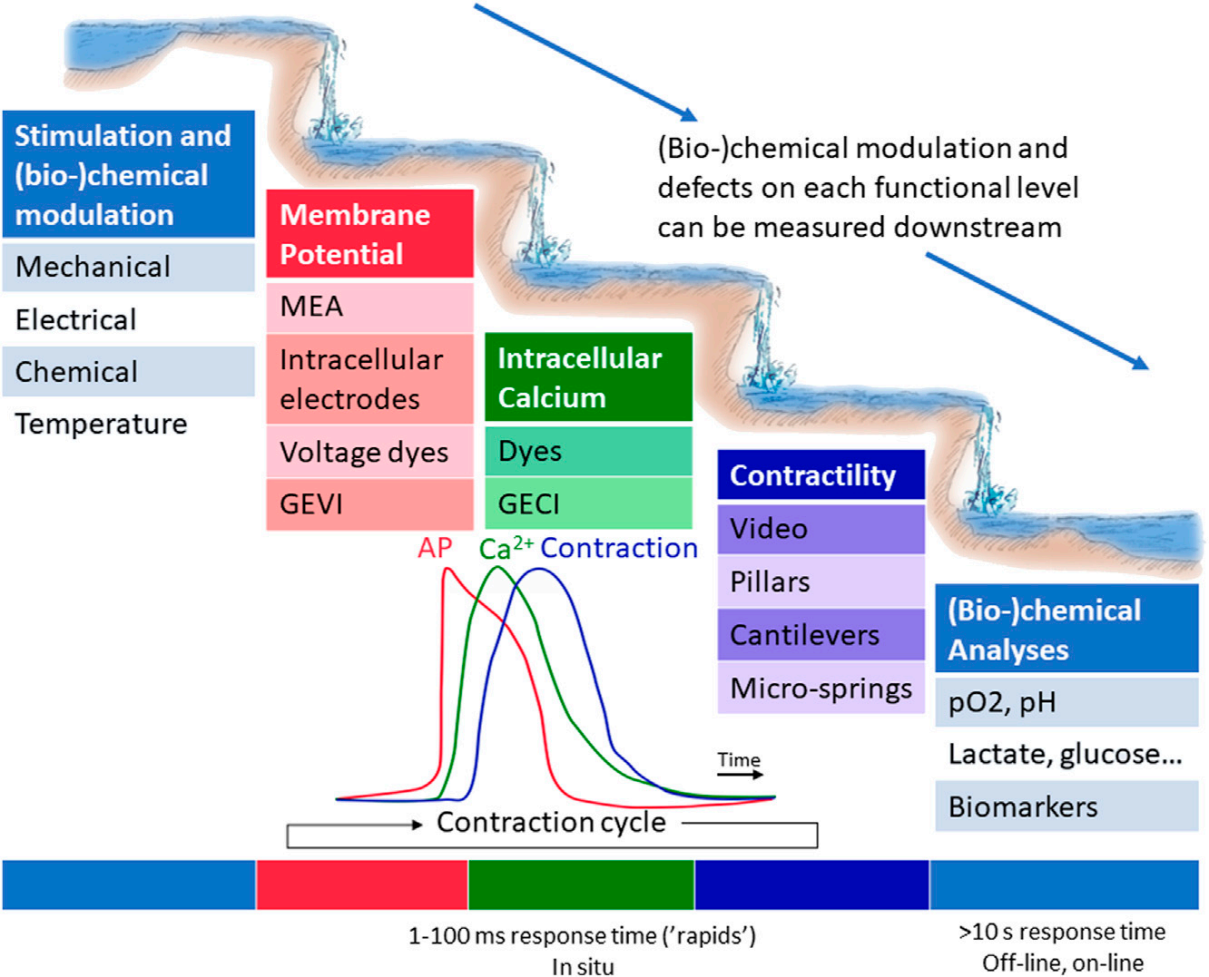
A functional scheme of a cardiac MPS. Various stimuli affect the cell block located in the rapid section, where the membrane potential gives rise to intracellular calcium flux, which in turn starts the mechanical contraction. The immediate functional responses can be recorded by measuring membrane potential, intracellular calcium or contractility via various *in situ* methods. (Bio-)chemical responses can typically be performed in-line, on-line or off-line.

The functional input-output scheme of a cardiac MPS can be symbolically described as a lake-rapids-lake section, depicted in Figure 5. The heart cell block is represented by the three middle plateaus and rapids sections, where the membrane potential gives rise to intracellular calcium flux, which in turn starts the mechanical contraction. These cell block functions are modulated by the various stimuli and (bio-)chemical changes entering the cell block from the lake above. All methods to measure membrane potential, calcium flux or the contractility can be placed under the heading “measurements of the cardiomyocyte contraction cycle”. These measurements must be carried out *in situ* and with fast enough sensors to detect the physiological signals in millisecond range. In addition, cardiac MPS respond biochemically by changing their metabolic state or by secreting biomarkers. Typically, (bio-)chemical responses can be measured in-line, on-line or off-line, and the response times are longer, typically over 10 s. All methods to measure these (bio-)chemical compounds can be placed under the title “(bio-)chemical measurements”.

Recently, many comprehensive reviews have been composed on the suitable sensor technologies for MPS systems, either aiming for a general overview (Modena et al., 2018; Young et al., 2019; Clarke et al., 2021; Sabaté del Río et al., 2023), or focusing on particular technological subsets such as biosensors (Senutovitch et al., 2015; Kratz et al., 2019; Ferrari et al., 2020; Mou et al., 2022; Rothbauer and Ertl, 2022), or electro-optical technologies (Kavand et al., 2022) or technical challenges in sensor fabrication and integration (Fuchs et al., 2021). Cardiac MPS–being electrically, mechanically and biochemically active–provides an attractive field for many complementing sensor technologies and, as such, would deserve a dedicated review paper. However, such sensor reviews focusing solely on cardiac MPS are rare; in fact, to our knowledge, the only recent review focusing solely on the cardiac MPS is the biosensor technology review by Criscione and co-workers (Criscione et al., 2023). In the following sections, we aim to provide a focused overview on the latest developments of the measurement solutions with cardiac MPS. Our aim is to illustrate the significance of the sensing building block of cardiac MPS and provide the reader an up-to-date list of further references.

The following survey focuses on cardiac MPS studies including perfusion, and new measurement technologies with potential for future MPS studies, with special emphasis on hiPSC-CMs. Our sensor review does not include the detection of structural and morphological changes or molecular analysis based on sequencing technologies, but an interested reader is invited to study the recent review of Arslan et al (Arslan et al., 2022).

### 6.1 Measurements of cardiomyocyte contraction cycle

The measurements of CM contraction cycle quantify the CM model functionality and acute responses to environment changes. In short, the CM contraction cycle begins with an AP, rapid depolarization of the membrane, which causes an inflow of Ca^2+^ ions. Due to the depolarization and calcium-induced calcium release, more Ca^2+^ is released from sarcoplasmic reticulum. The binding of Ca^2+^ to troponin C initiates cellular contraction. Thus, the contraction cycle can be roughly characterized on three levels, as illustrated in the Figure 5 analogue: measurements of electrophysiological properties, measurements of intracellular calcium, and measurements of contractility and biomechanics. The rapid processes of contraction can be illustrated as a series of waterfalls, each influencing the flow in subsequent waterfall. On the other hand, structural defects may not manifest themselves as disturbances on the electrical level. The waterfalls require measurements with high sampling rates *in situ*, whereas the (bio-)chemical changes in the culture are slower, and can thus be measured in the calm lakes, accumulations of several contractions.

An advantage of heart MPS is the capability to include multiple sensors and electrodes for continuous measurement and monitoring of multiple measurement modalities, even concurrently. However, the system itself, and the tissues being measured present challenges to the traditional *in vitro* measurement techniques, with aspects such as long-term measurements and the inclusion of medium perfusion. The inclusion of vascular-like perfusion in MPS studies is an important aspect of experiment design due to its physiological analogues and its use in pharmacological studies. Further, it enables the long-term quantification of functionality and improves the model quality, such as Ca^2+^ transient amplitude increase and time-to-peak duration decrease (King et al., 2022). Here, the following sections and Table 5 briefly review these methods, their uses cases and discuss possible prospective technologies in MPS taking the previously mentioned aspects into consideration.

**TABLE 5:** Summary of recent studies with perfusion and measurements of contraction cycle in hiPSC-CMs, and recently developed technologies with potential to be use in MPS studies.

Article	Cell type(s)	Electrical	Calcium	M	V	P	Notes
Perfusion, hiPSC-CMs							
Bergström et al. (2015)	hiPSC-CM				**x**	x	
Mathur et al. (2015)	3D - hiPSC-CM		GCaMP3		**x**	x	
Zhang et al. (2016)	3D - hiPSC-CM, primary human hepatocytes, hepatocellular carcinoma				**x**	x	
Zhang et al. (2016)	Rat, HUVEC, hiPSC-CM				**x**	x	
Ellis et al. (2017)	3D - hiPSC-CM, endothelial	MEA	Fluo-4 AM			x	
Maoz et al. (2017)	hiPSC-CM, HUVEC	MEA				x	
Skardal et al. (2017)	3D - hiPSC-CM, liver, lung				**x**	x	
Schneider et al. (2019)	3D - hiPSC-CM, rat				**x**	x	OpenHeartWare software
Chramiec et al. (2020)	3D - hiPSC-CM, MSC, Ewing Sarcoma		Fluo-4		**x**	x	
Lee et al. (2021)	hiPSC-CM, fibroblasts, myofibroblasts, breast cancer spheroids	MEA				x	
Visone et al. (2021)	hiPSC-CM, Neonatal rat	**x**				x	Micro-electrode guides
King et al. (2022)	3D - hiPSC-CM, microvascular endothelial, fibroblast		GCaMP6f		**x**	**x**	MUSCLEMOTION software
Schneider et al. (2022)	3D - hiPSC-CM, primary human dermal fibroblasts				**x**	**x**	OpenHeartWare software
Perfusion, no hiPSC-CMs							
Liu et al. (2020a)	Mouse HL-1	**x**				**x**	Extracellular, and intracellular nanopillar electrodes
Mozneb et al. (2020)	Neonatal rat				**x**	**x**	Surface plasmon resonance measurement
Sakamiya et al. (2020)	3D - Rat CM		Fluo-4 AM	x		**x**	Piezoelectric Micro-pillar array
No perfusion							
Oleaga et al. (2016)	hiPSC-CM, HepG2/C3A, skeletal myofibers, motoneurons			**x**	**x**	-	Microscale cantilevers
Wang et al. (2018)	3D - Rat CM		Fluo-8 AM	x		-	Integrated micro-springs
Desbiolles et al. (2019)	Rat CM			**x**		-	Nanovolcanoes
Abulaiti et al. (2020)	3D - hiPSC-CM		GCaMP3	x	**x**	-	Microelectromechanical measurement system
Faulkner-Jones et al. (2022)	3D - hiPSC-CM	Di-4-ANEPPS	Fura 4-AM		**x**	-	CellOptiq software

CM, Cardiomyocyte; hiPSC, human induced pluripotent stem cell; HUVEC, human umbilical vein endothelial cells. Column labels: M, Mechanical; V, Video; P, Perfusion.

Our review suggests that there are emerging technologies for mechanical and electrical measurements, which are likely to be adopted in the future. The efficacy of the most novel methods have yet to be evaluated in hiPSC-CM disease models and drug studies. The reviewed studies show the potential of combining different measurement modalities, which may be fruitful for perfusion-enabled heart-on-chip studies as the field matures.

#### 6.1.1 Electrodes

While intracellular AP recordings have traditionally been the gold standard of *in vitro* electrophysiology, patch clamp is not a practical option in MPS as would require extracting the cells from the chip and is not well suited for long term measurements. Recent advances in micro- and nanotechnology have enabled long-term intracellular measurements, providing characterization of action potential -resembling signals Desbiolles and colleagues (Desbiolles et al., 2019) demonstrated the use of volcano-shaped nanostructure, which were capable of recording typical AP shapes and associated characteristics, matching those of patch clamp, with the exception of attenuated AP amplitudes. Similarly, Liu and colleagues demonstrated nanopillar electrodes (Liu H. et al., 2020) in hypoxia measurements and obtained AP-like readouts. Visone and colleagues introduced micro-electrode coaxial channel guides (Visone et al., 2021), to characterize drug-induced electrophysiological changes. In contrast to patch clamp, their setup guides tissue formation to accessible measurement sites. This also enables future studies in 3D cultures using the setup.

On a larger scale of cultures, microelectrode arrays are an accessible option, capable of measuring the field potential in culture without perforating the cell membrane. While they do not provide as accurate electrical information as intracellular measurements, the instrumentation requirements are lower and enable modular designs. They also present an advantage in being able to track the electrical propagation in the culture. While the MEA technology has developed largely in context of 2D cultures, techniques such as inkjet-printed 3D pillar electrodes have enabled 3D MEA designs as well (Grob et al., 2020). Further, there are multiple software tools available for the signal processing (Pradhapan et al., 2013; Dunham et al., 2022) and MEA presents a well-established means of measurement. The parametrization of APs and field potentials has been well established, enabling clear comparison of results between studies (Kussauer et al., 2019).

In studies involving characterization of diseases in heart-on-chip models and the need for understanding electromechanical coupling, electrical measurements and/or calcium imaging has been included, with MEAs being a typically used means of electrical measurement (Mathur et al., 2015; Ellis et al., 2017; Maoz et al., 2017; Chramiec et al., 2020; Lee et al., 2021).

#### 6.1.2 Indicators

Voltage- and calcium-sensitive dyes enable high-resolution analysis of the AP propagation and calcium fluxes, respectively. Using these indicator dyes, such as di-8-ANEPPS for voltage and Fluo-4 for calcium, stresses the cells and affects the cellular function (Douthwright and Sluder, 2017). Thus, the method is not well suited for long-term measurements (O’Shea et al., 2020). Some of these disadvantages have been overcome by genetical encoding of fluorescent proteins. Genetically encoded voltage indicators (GEVI) present lower phototoxicity and can thus be measured repeatedly. They do, however, exhibit slower response times when compared to loaded dyes (Leyton-Mange et al., 2014). Similarly, genetically encoded calcium indicators (GECI) such as GCaMP3 have presented new alternatives for traditional Ca^2+^ sensitive dyes (Zarowny et al., 2020). While these indicators have been primarily used in 2D cultures, studies in 3D cultures have been performed recently (Dingle et al., 2020). In contrast to traditional dyes, GECI can be targeted to specific cell types and the measurement repeatability enables its use, e.g., for monitoring of maturation (Shinnawi et al., 2015). However, GECI can interfere with cellular calcium signaling by buffering native Ca^2+^ (Tian et al., 2009). While these indicators can be used individually, combined constructs have been developed and used as well (Hou et al., 2014; Björk et al., 2017).

#### 6.1.3 Contractility

MPS designs typically incorporate capability for optical transmission. Thus, video-based measurements can provide a view of the contraction cycle. When moving towards more complex CM structures, microfabricated structures become more feasible due to the limitations of light transmission.

New methods for quantifying hiPSC-CM model contractility have enabled studies of their biomechanical phenotype. Further, with more complex CM models being developed, specific setups for dissociated cells, monolayers and engineered heart tissue are needed. Here, we briefly cover the main contractility-based assays and their operating principles, as contractility-based measurements have been recently reviewed in detail by Dou and colleagues (Dou et al., 2022).

Traditionally, atomic force microscopy and traction force microscopy have been used for measuring CM biomechanics. Due to their requirements in instrumentation and expertise, their use cases in MPS are limited. Novel methods involving microscale structures and video-based measurements have become a more accessible ways of characterizing contractility due to their instrumentation requirements and capability of long-term measurements, enabling their use in heart MPS.

Microfabricated structures have the capability of directly measuring cellular contraction force, while minimally affecting the cell culture. Setups such as piezoelectric micropillar arrays (Sakamiya et al., 2020), microcantilevers (Oleaga et al., 2016; Turnbull et al., 2021) and micro-springs (Wang et al., 2018) represent recent advances in direct measurements of cellular contraction force, which minimally affect the cell culture. Impedance-based measurement of contraction presents another option for indirect force measurement (Wang et al., 2020). The strategies of integrating mechanical sensing assays in microfluidics-capable organ-on-chips have recently been reviewed by Morales and colleagues (Morales et al., 2022).

Transmission video microscopy -based quantification of contraction involves methods that measure the displacement of edges, the intensity of pixels, and correlation-based block matching. Different implementations of the method have been introduced by various groups (Ahola et al., 2014; Huebsch et al., 2015; Sala et al., 2018; Schneider et al., 2019). Due to its straightforward instrumentation–from brightfield microscopy to digital holographic microscopy (Jaferzadeh et al., 2020) – it requires only a microscope and a camera and has thus been used in numerous MPS studies, as evidenced by its prevalence in the following review of recent publications. Transmitted light microscopy-based measurements are typically restricted by the transparency and thickness of the sample, limiting their use in more complex organ-on-chip models. Further, they are not capable of measuring the vertical contractility, but axial and vertical force are strongly correlated (Turnbull et al., 2021).

Most studies involving perfusion use optical video measurements as their primary model of quantification. In studies that include multiple cell types and complex 3D structures (Zhang et al., 2016; Skardal et al., 2017; Schneider et al., 2022) or where the main aim has been the demonstration of new related technologies (Bergström et al., 2015; Schneider et al., 2019), video-based quantification has provided an accessible way of quantifying CM cultures. Video-based measures may provide resistance to perfusion-induced noise in the quantification, explaining its prevalence in the reviewed studies.

### 6.2 (Bio-)chemical measurements

In a proper MPS, the environmental conditions should mimic the corresponding conditions *in vivo*. In essence, temperature and pH should be kept within physiological values and, importantly as well, partial oxygen pressure (pO_2_) should be regulated within tissue-specific limits (Wilson, 2008). In addition, MPS systems modelling cardiac ischemia reperfusion should provide means to generate ischemic events on demand. For the pO_2_ regulation and control purposes alone, MPS systems should contain means for real time pO_2_ monitoring. Moreover, as described earlier the monitoring of pO_2_ and other chemical and biochemical parameters provides important functional data about the metabolic and systemic state of the system. The following two subchapters and Table 6 gives a brief survey on the most important chemical analytes for cardiac MPS and available measurement technologies.

**TABLE 6:** Summary of recent heart-on-chip studies with (bio-)chemical monitoring.

Article	Cell type	Oxygen	pH	Biosensing		Perfusion
Schneider et al. (2022)	3D hiPSC-CMs, primary human dermal fibroblasts	Luminescence, fiberoptic			*in situ*	x
Zhang et al. (2017)	3D Human primary hepatocytes, human iPSC-CMs	Luminescence	optical absorption	Electrochemical immu-nosensor: CK-MB	in-line	x
Tanumihardja et al. (2021)	2D hPSC-CM s	RuOx electrode, potentiometric	RuOx electrode, chronoamperometric		*in situ*	static
Lee et al. (2021)	3D iPSC-CMs, brest cancer tissues	-	-	electrochemical immuno-aptasensing; Troponin T, CK-MB, HER-2, IL-8	on-line	x
Shin et al. (2016)	ESC-CMs			Electrochemical immunoaptasensing; CK-MB	off-line	x
Kolanowski et al. (2020)	hiPSC-CMs	Luminescence, fiberoptic			in-line	x
Häkli et al. (2022b)	hiPSC-CMs monolayer	Luminescence			*in situ*	static
Häkli et al. (2021)	hiPSC-CMs monolayer	Luminescence			*in situ*	static

#### 6.2.1 Oxygen

Overall, pO_2_ is by far the most common chemical parameter monitored in cardiac MPS systems. It is the essential parameter defining the microenvironment, reflects the metabolic status and plays a key role in ischemic cardiac models.

Molecular oxygen concentration affects cardiac metabolism, cell growth, differentiation and cell signaling. Vice versa, the number of cells and their growth rate as well as their metabolic status affect the local oxygen consumption and concentration. In a human heart, normal physiological pO_2_ is strongly heterogenous, varying from 2.0 kPa found in parts of myocardium up to 12.5 kPa in pulmonary veins (Keeley and Mann, 2019). Many MPS chips contain oxygen-permeable structures made of materials such as PDMS, which further contribute to the local dynamics and heterogeneity of the oxygen distribution. Therefore, it is important to have means for localized real-time measurement and control of oxygen. Indeed, the oxygen monitoring should take place *in situ* and preferably in multiple locations in 3D, which presents a great challenge (Place et al., 2017). Comprehensive reviews on current methodologies for oxygen measurement and control in MPS systems have recently been composed by Rivera and co-workers (Rivera et al., 2019) and earlier by Oomen et al (Oomen et al., 2016).

Electrochemical methods and optical methods based on luminescence quenching and electrochemical methods enable *in situ* oxygen monitoring (Papkovsky and Dmitriev, 2013). The key advantages of electrochemical oxygen sensors include linear behavior, typically a one-point calibration method and compatibility of sensing electrode preparation with lithographic manufacturing processes. However, these sensors usually consume oxygen, and imaging or scanning set-ups are challenging to realize (Wolfbeis, 2015). Optical methods, on the other hand, are typically minimally invasive, do not consume oxygen and are compatible with imaging set-ups both in 2D and 3D. Many commercial devices based on optical methods exist, and comprehensive technology reviews are available (Wang and Wolfbeis, 2014). However, despite the importance of the analyte and available technologies, proper cardiac MPS with *in situ* oxygen monitoring are still relatively rare and typically only demonstrative.

Recently, Schneider and co-workers presented an interesting work on hiPSC-CM-based cardiac micro tissues inside a microfluidic system equipped with an electrical spacing system (Schneider et al., 2022). Based on their previous works on the luminescent-based chemical sensing in microfluidic chips (Zirath et al., 2018; Müller et al., 2021; Zirath et al., 2021), they integrated *in situ* oxygen sensing into their system and demonstrated the positive correlation between the pacing frequency and the local oxygen consumption rate. Zhang and co-workers reported on an MPS system with in-line and on-line sensing platforms (Zhang et al., 2017). The system can contain multiple organs connected through perfusion channels, equipped with in-line sensor modules to monitor temperature, pO_2_ and pH. In addition, an on-line biochemical sensing module can be activated by opening a valve on demand and used for monitoring of biomarkers. The system was applied in demonstrative drug response and toxicity studies with human heart-and-liver-on-chip and human heart-and-liver-cancer-on-chip models.

Kolanowski and co-workers showed recently that the structural maturation of hiPSC-CMs can be enhanced through cyclic pulsatile hemodynamic forces during the perfusion (Kolanowski et al., 2020). In addition, the tailored microfluidic platform contained means for oxygen control and a luminescence-based commercial oxygen sensors placed up- and downstream from the cell culture chamber.

While the number of studies with 3D cardiac tissues under a continuous perfusion and with integrated (bio-)chemical monitoring is still very limited, the number of studies in static 2D/3D models in substantially greater. Tanumihardja and co-workers applied ruthenium oxide (RuOx) electrode in two different modes to monitor the oxygen concentration and pH of static 2D hiPSC-CM culture in real time (Tanumihardja et al., 2021). Häkli and co-workers have recently studied the electrophysiological responses of hiPSC-CMs under ischemia and reperfusion (Häkli et al., 2021; 2022b), where they applied the luminescence-based oxygen sensing with a tailored in-contact parabolic lens (Välimäki et al., 2020).

Compared to pO_2_, the real-time monitoring of other (bio-)chemical parameters is still in very early stages and reports are rare. A probable reason for this is the abundance of means for the measurement of contraction cycle, providing high-quality functional data rapidly and sensitively responding to stimuli and changes in the culturing conditions. Nevertheless, metabolic parameters and biomarkers are important, and the following aims to summarize the recent studies with this regard.

#### 6.2.2 pH and cardiac biomarkers

In addition to the pO_2_, parameters such as pH, glucose and lactate provide information about the culture conditions and metabolic status of the system. Indeed, two of the above-mentioned studies with oxygen monitoring contained in-line pH monitoring by electrochemical means as well (Zhang et al., 2017; Tanumihardja et al., 2021). In both cases, the pH monitoring was utilized to the ensure stable and neutral pH environment during the studies.

Furthermore, Zhang and co-workers applied their on-line biochemical sensing module to monitor the biomolecule secretion during the studies of the heart-and-liver- and heart-and-liver-cancer-on-chips. The module was able to generate data about albumin and glutathione S-transferase production of the liver organoids as well as creatine phosphokinase-MB (CK-MB) production of cardiac organoids with high enough sensitivity for over 5 days. Lee and co-workers have reported on impressive studies about the cardiotoxicity of cancer chemotherapy (Lee et al., 2021), combining breast cancer spheroids with hiPSC-derived cardiac spheroids on a dual organ-on-a-chip platform with integrated on-line electrochemical immunoaptasensing module. Cardiac markers Troponin T, CK-MB as well as cancer growth related human epidermal growth factor (HER-2) were successfully monitored over 5 days.

Interestingly, we were not able to find any reports on cardiac MPS with on-line glucose or lactate monitoring. While electrochemical glucose and lactate sensing are relatively widely applied, for example, drug screening in cancer research, it has so far attracted only little interest among cardiac researchers.

## 7 Computational modelling and simulation

The roles of computer modelling and simulation (M&S) in the cardiovascular field are manifold, from basic science to translational research and from pharmaceutical development to clinical medicine (for reviews, see, e.g., (Colatsky et al., 2016; Niederer et al., 2019; Koivumäki et al., 2022)). Models offer essential tools to generate hypothesis, integrate and interpret data, and to further mechanistic understanding of physiological processes. These use cases are undoubtedly similarly relevant and potential in the context of cardiac MPS. From the computer M&S perspective, MPS offers exciting new data avenues owing to overall observability and controllability of the system. More specifically, the capability for continuous/long-term and multimodal measurements and concurrent sensing of the central functional readouts, as reviewed above in chapter 6.1, generate unprecedently holistic dataset for parameterization and validation of computational models.

In this chapter, we will first review the nuts and bolts of *in silico* MPS that are the biophysical models of hiPSC-CM function and the approaches developed to model cell-cell interactions. Then, we will the discuss computational modelling as a tool for 1) interpreting the highly variable *in vitro* results from different laboratories, and 2) translating the hiPSC-based findings to the human context. Finally, we will summarize the current state of the art of replicating MPS *in silico*.

### 7.1 Replicating electromechanical phenotype of hiPSC-CMs *in silico*

Computational models of hiPSC-CM ion dynamics and electrophysiology have been developed since 2012, and their pedigree has diverged to five unique branches. They do, however, share a basis that is common to all biophysics-based models of AP in cardiomyocytes: Hodgkin and Huxley (Hodgkin and Huxley, 1952) formalism to describe ion currents and fluxes. The shared and unique characteristics of the five pedigree branches of hiPSC-CM models are described in detail in Table 7.

**TABLE 7:** Summary of hiPSC-CM models’ features, including electrophysiology, calcium dynamics, and biomechanics.

Initial Public	hiPSC-specificity	Electrophysiology	Calcium dynamics	BM
Zhang et al. (2012a)	“hiPSC-like” model	based on (ten Tusscher et al., 2004) hV-CM model	based on (ten Tusscher et al., 2004)	no
	+ membrane clock	added I_f_ and I_NaL_	no spontaneous SR Ca^2+^ release	
	- no calcium clock	I_CaL_, I_Kr_, and I_K1_ conductances altered to obtain AP morphology typical for hiPSC-CMs		
Lemoine et al. (2018)	“hiPSC-like” model	based on (Grandi et al., 2010) hV-CM model	unmodified from (Grandi et al., 2010) hV-CM model	no
	- no membrane clock	added I_CaT_	no spontaneous SR Ca^2+^ release	
	- no calcium clock	I_CaL_, I_Kr_, and I_K1_ conductances adjusted to match *in vitro* hiPSC-CM data		
Prondzynski et al. (2019)	“hiPSC-like” model	same as (Lemoine et al., 2018)	same as (Lemoine et al., 2018)	yes
	- no membrane clock			
	- no calcium clock			
Paci et al. (2013)	hiPSC-CM model	based on (ten Tusscher et al., 2004) hV-CM model	modified from (ten Tusscher et al., 2004) to account for lack of t-tubuli	no
	- unphysiological membrane clock, based on Na+ window current	added I_f_	no spontaneous SR Ca^2+^ release	
	- no calcium clock	extensive reparameterization of Na^+^, Ca^2+^ and K^+^ currents based on *in vitro* hiPSC-CM data		
Paci et al. (2018)	hiPSC-CM model	based on (Paci et al., 2013) hiPSC-CM model	reformulated SR Ca^2+^ release based on (Koivumäki et al., 2011) hA-CM model	no
	- unphysiological membrane clock, based on Na+ window current		spontaneous SR Ca^2+^ release	
	+ calcium clock			
Paci et al. (2020)	hiPSC-CM model	based on (Paci et al., 2013) hiPSC-CM model	based on (Paci et al., 2018)	no
	+ membrane clock	adopted I_Na_ and I_f_ from (Koivumäki et al., 2018) hiPSC-CM model	spontaneous SR Ca^2+^ release	
	+ calcium clock			
Forouzandehmehr et al. (2021), 2022	hiPSC-CM model	based on (Paci et al., 2020)	based on (Paci et al., 2020)	yes
	+ membrane clock		spontaneous SR Ca^2+^ release	
	+ calcium clock			
Koivumäki et al. (2018)	hiPSC-CM model	based on (Paci et al., 2015) hiPSC-CM model	modified from (Korhonen et al., 2010) to account for the immature hiPSC-CM ultrastructure	no
	+ membrane clock	reparameterization of Na^+^ and Ca^2+^ currents based on *in vitro* hiPSC-CM data	spontaneous SR Ca^2+^ release	
	+ calcium clock			
Kernik et al. (2019)	hiPSC-CM model	reformulated of Na^+^, Ca^2+^, and K^+^ currents based on *in vitro* hiPSC-CM data	full parameterization of based on *in vitro* hiPSC-CM data	no
	+ membrane clock		no spontaneous SR Ca^2+^ release	
	+ calcium clock (partial)			
Jæger et al. (2020)	hiPSC-CM model	based on (Grandi et al., 2010) and (O’Hara et al., 2011) hV-CM models, and Paci et al. hiPSC-CM models	modified from (Grandi et al., 2010)	no
	- no membrane clock		no spontaneous SR Ca^2+^ release	
	- no calcium clock			

BM, Biomechanics; I_f_, pacemaking current; I_Na_ and I_NaL_, fast and late Na^+^ current; I_CaL_ and I_CaT_, L-type and T-type Ca^2+^ current; I_Kr_, delayed rectifier K^+^ current; I_K1_, inward rectifier K^+^ current; hV-CM, human ventricular cardiomyocyte; hA-CM, human atrial cardiomyocyte.

In both low and high throughput *in vitro* studies of hiPSC-CMs, the central readouts were initially the AP and the CT. Indeed, analysis of the (rather) standardized morphological biomarkers of these two variables provides a good characterization of the electrophysiological phenotype, while also alluding towards the bigger picture of the excitation-contraction coupling phenotype. Building on that tradition, during the past few years, contractile force and its dynamics have gained an equally important role as a measurand in hiPSC-CM studies.

The developmental steps of computational hiPSC-CM models have paralleled the progress in experimental procedures. Initially, the emphasis was in modeling of electrophysiology and intracellular ion dynamics (see Table 7 for details). Recently, hiPSC-CM models have been extended to include mathematical description of contractile cellular mechanics. Actually, pioneering research was published by Frotscher and colleagues already in 2015 and 2016 (Frotscher et al., 2015; Frotscher et al., 2016). As the authors’ focus was to develop an electromechanically coupled model for EHT, they used a purkinje fibre CM model (McAllister et al., 1975) to recapitulate the immature phenotype of hiPSC-CM and coupled that cellular electrophysiology model with the contractility component (Hunter et al., 1998). We have recently published the first hiPSC-CM-specific electromechanical models (Forouzandehmehr et al., 2021; Forouzandehmehr et al., 2022).

### 7.2 Modelling as a translational tool

As discussed in chapter 3, although there is continuous development of differentiation methods, the hiPSC-CMs phenotype differs from the native human counterparts, having some foetal features. In this context, computational modelling offers essential translational tools. Indeed, those capabilities have already been demonstrated in multiple independent studies, in which *in vitro* hiPSC-CM-based findings were translated to the human context (Gong and Sobie, 2018; Koivumäki et al., 2018; Tveito et al., 2018; Jæger et al., 2020). Accessibility and applicability of such tools would greatly benefit from transparent comparisons of the computational models, as was done recently for hiPSC-CM models by (Paci et al., 2021).

Capabilities of the so-called *in silico* drug trials in early-phase cardiotoxicity detection have already been demonstrated in that human ventricular CM models to have higher accuracy than *ex vivo* animal models in predicting clinical risk of drug-induced arrhythmias (Passini et al., 2017). The physiological robustness and specificity of computational hiPSC-CM models is approaching the needed level, so that they serve a corresponding role in high-throughput analysis of drug candidates.

### 7.3 Cell-cell interaction, tissue function, and MPS

The technical concepts for utilization of hiPSC-CM-based *in vitro* system stretch from single CMs, CM clusters, and 2D monolayers to organoids, and all the way to EHT. While the more environmentally complex end of the spectrum replicates to some extent *in situ* cell-cell communications, in 2D monolayers and CM clusters even the basic electrophysiological and biomechanical cell–cell interactions have unique properties. To recapitulate the conduction of electrical and mechanical activation in such an MPS system, the classical homogenization/continuum-based approaches of modelling tissue/organ function are likely to fall short. Instead, approaches that explicitly represent the extra- and intracellular spaces as well as cell–cell connections will be essential to capture the heterogeneities (for an extensive review, see (Tveito et al., 2021)).

To our knowledge, multiphysics models that would put together all the building blocks of MPS in integrative simulations have not yet been published. First steps towards using a classical continuum-based fully coupled electromechanical model for MPS were taken by (Frotscher et al., 2015; Frotscher et al., 2016). More recently, the same group extended their tissue model to include also fibroblasts in addition hiPSC-CMs, thus enabling mechanistic analysis of their contribution to contractility in MPS (Jung and Staat, 2019). Expanding the models to include vasculature and innervation is an interesting but not trivial step. Also, as preliminary studies of replicating environmental control and physical stimuli of MPS in simulations have been teased in conference presentation, those building blocks are bound to see light of the day in the not-too-distant future.

## 8 Outlook

**FIGURE 6:**
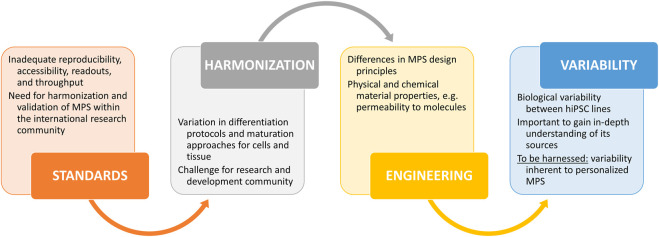
Challenges and opportunities related to the MPS building blocks and their integration.

In this review, we chose heart as an example in development of MPS and described the state-of-the art building blocks including cardiac cell types, hydrogels, mechanical stimulation, essential functional measurements, and computational modeling as translational tool. Although MPS are emerging tools to study cardiac (patho)physiology, some of the building blocks are still in their infancy. Although vascularization is implemented in several cardiac applications only few MPS include innervation especially in more complex 3D environment. Furthermore, combination of the two network systems as transportation and control components should be considered when aiming for more mature MPS. Figure 6 represents opportunities as well as challenges related to MPS and their integration.

Multidisciplinary expertise is required to produce mature biological, technical, and computational components and, finally, combine them into functional MPS or even Body-on-Chips. Due to improved capability to capture human physiological responses, MPS are an attractive option not only in academic research but also in drug discovery and regulatory testing. However, probably several different types of *in vitro* test systems holding varying level of complexity from 2D monocultures to tissue mimicking MPS are needed for future safety and efficacy assessment of compounds. Computer M&S offer potential tools to compare the results obtained with the different systems. Interestingly, a paradigm shift towards *in vitro* and *in silico* -based safety and efficacy assessment was recently established in FDA’s Modernization Act 2.0 stating that mandatory animal experiments are no longer required in drug development before entering into clinical trials. Recent development in more complex and predictive test systems such as MPS, organoids and computational models has backed up the FDA’s modernization act, and other revisions in regulatory guidelines may be on their way. These statements highlight the need for MPS and call for validated and standardized test systems with high throughput capacity.

## Footnotes

1
https://www.fda.gov/science-research/about-science-research-fda/advancing-alternative-methods-fda [Accessed 27 April 2023].
